# Supplementary material for: Circulating tumour DNA sequence analysis as an alternative to multiple myeloma bone marrow aspirates
Source: Nat Commun. 2017 May 11;8:15086. doi: 10.1038/ncomms15086 (PMC5437268; doi:10.1038/ncomms15086)
Supplement: Supplementary Information — Supplementary Figures, Supplementary Tables, Supplementary Note, Supplementary Methods and Supplementary References [file ncomms15086-s1.pdf]

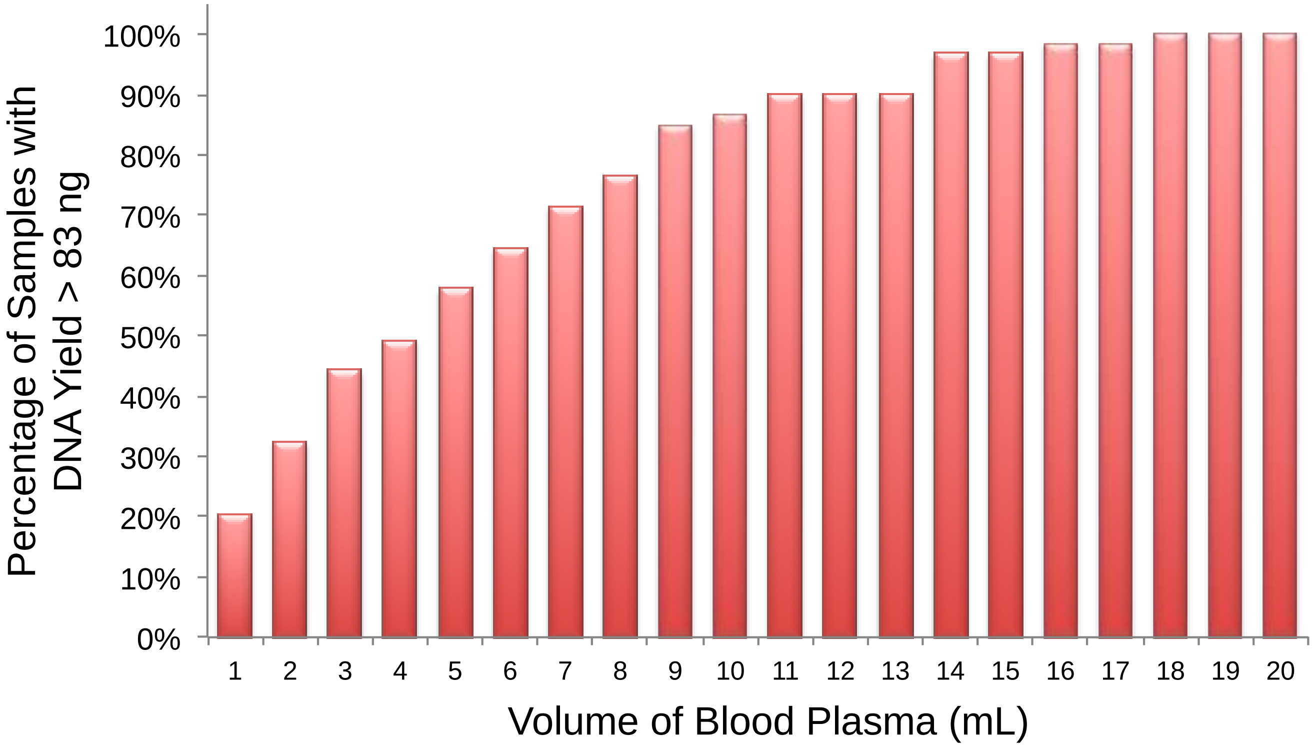

Supplementary Figure 1. Estimated volume of blood plasma needed to collect 83 ηg of cfDNA in MM. Using cfDNA concentrations in blood plasma obtained from 59 plasma samples from 48 MM patients included in this study, we estimated that 83 ηg of cfDNA would have been attained from all patients through collection of at least 18 mL of blood plasma. In this study, cfDNA was extracted from 3-13 mL of plasma resulting in insufficient cfDNA yields (34-80 ηg) in 11 of 59 samples (18.6%).

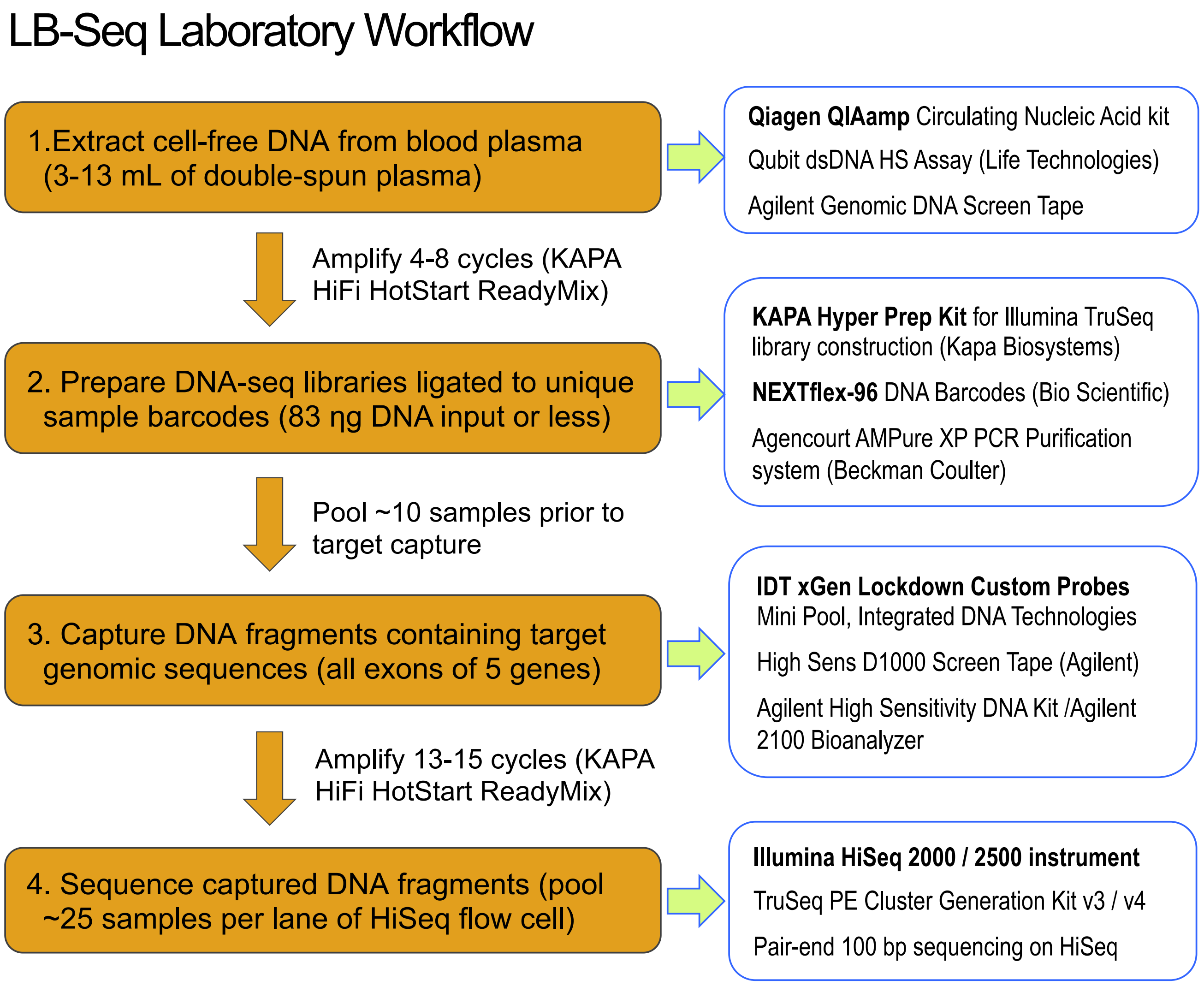

Supplementary Figure 2. LB-Seq laboratory workflow.

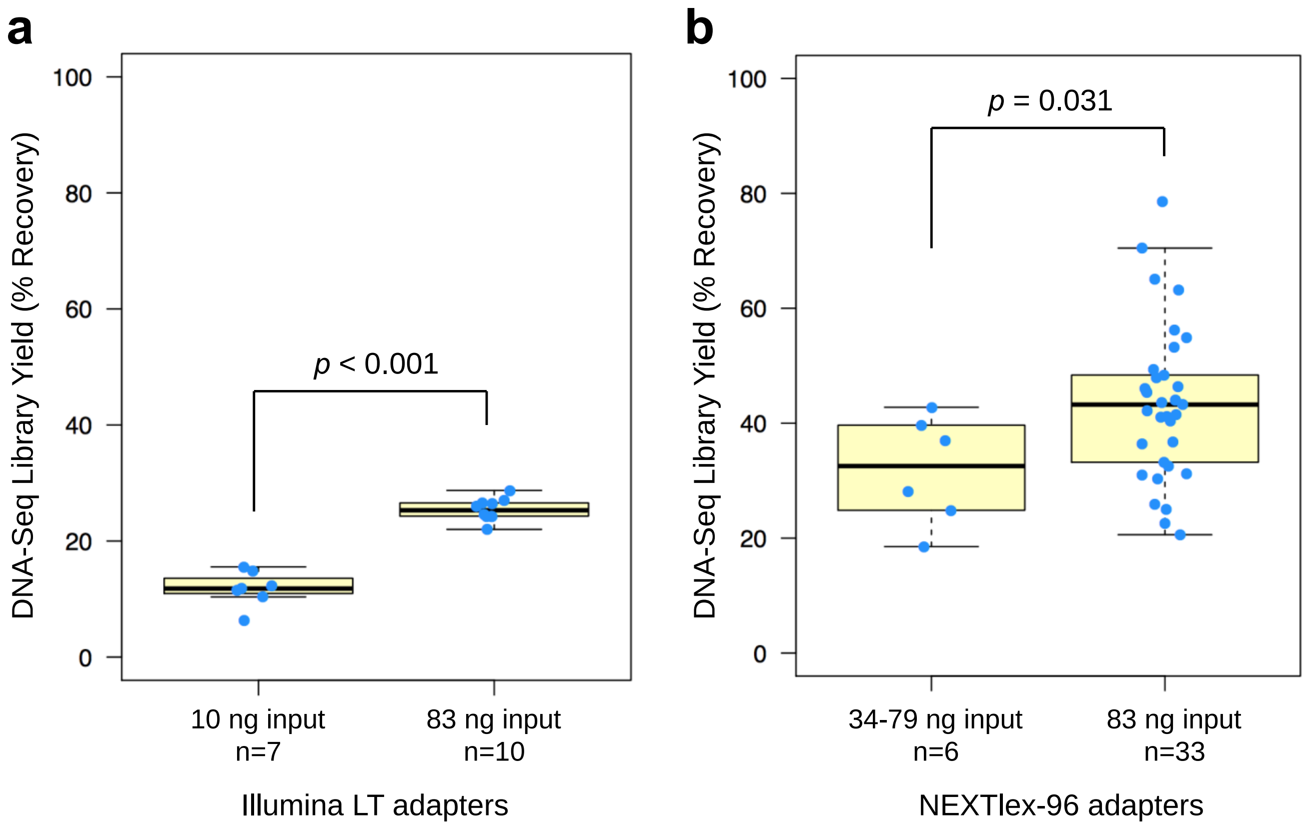

Supplementary Figure 3. Comparison of DNA-seq library construction efficiency using different sample barcode types and low or high cfDNA input. In the process of developing the method for cfDNA library construction, we tested two types of sample indexing adapters, Illumina TruSeq LT adapters (**a**), which included 6 nt and 8 nt indexes obtained from Illumina (San Diego, CA), and Illumina-compatible 8 nt index NEXTflex-96 DNA barcodes (**b**) obtained from Bioo Scientific (Austin, TX). Each plot represents the distribution of library yields, expressed as percent of theoretical yield, for separate cfDNA samples (no technical or biological replicates). The number of samples in each group indicated in the figure and each plot is overlaid with the corresponding box plot, where the central rectangle spans the first to the third quartile (interquartile range or IQR). A segment inside the rectangle shows the median, and "whiskers" above and below the box show the value 1.5×IQR above or below the third or the first quartile, respectively. Statistical analyses were performed using the Wilcoxon test with *P* value of 0.05 considered statistically significant.

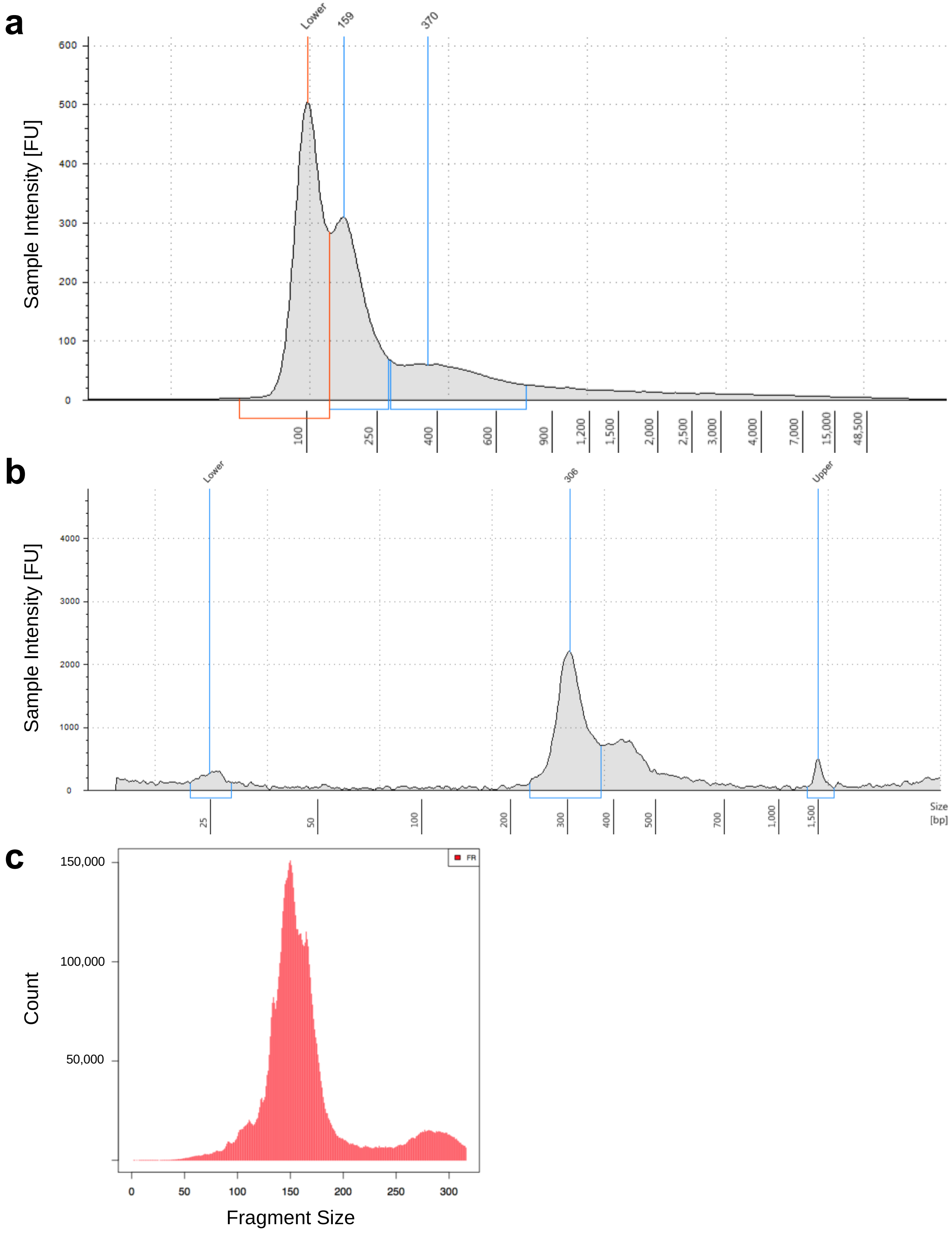

a
Lower
159
370
Sample Intensity [FU]
600
500
400
300
200
100
0
100
250
400
600
900
1,200
1,500
2,000
2,500
3,000
4,000
7,000
15,000
48,500
b
Lower
306
Upper
Sample Intensity [FU]
4000
3000
2000
1000
0
25
50
100
200
300
400
500
700
1,000
1,500
Size [bp]
c
Count
150,000
100,000
50,000
FR
0
50
100
150
200
250
300
Fragment Size

Supplementary Figure 4. DNA fragment size distribution in extracted cfDNA, DNA-seq libraries, and sequencing data. DNA fragment size distribution in extracted cfDNA sample (**a**) and adapter-ligated DNA-sequencing library (**b**) is compared to the inferred insert size of sequenced DNA fragments obtained from mapping of DNA sequencing reads (**c**). The major peak near 159 bp, corresponding to DNA fragment wrapped around one nucleosome, shifts to 306 bp following adapter ligation, and a similar peak (near 150 bp) is observed in the insert size distribution. Similar pattern is observed for a peak near 370 bp corresponding to DNA fragments wrapped around two-nucleosomes.

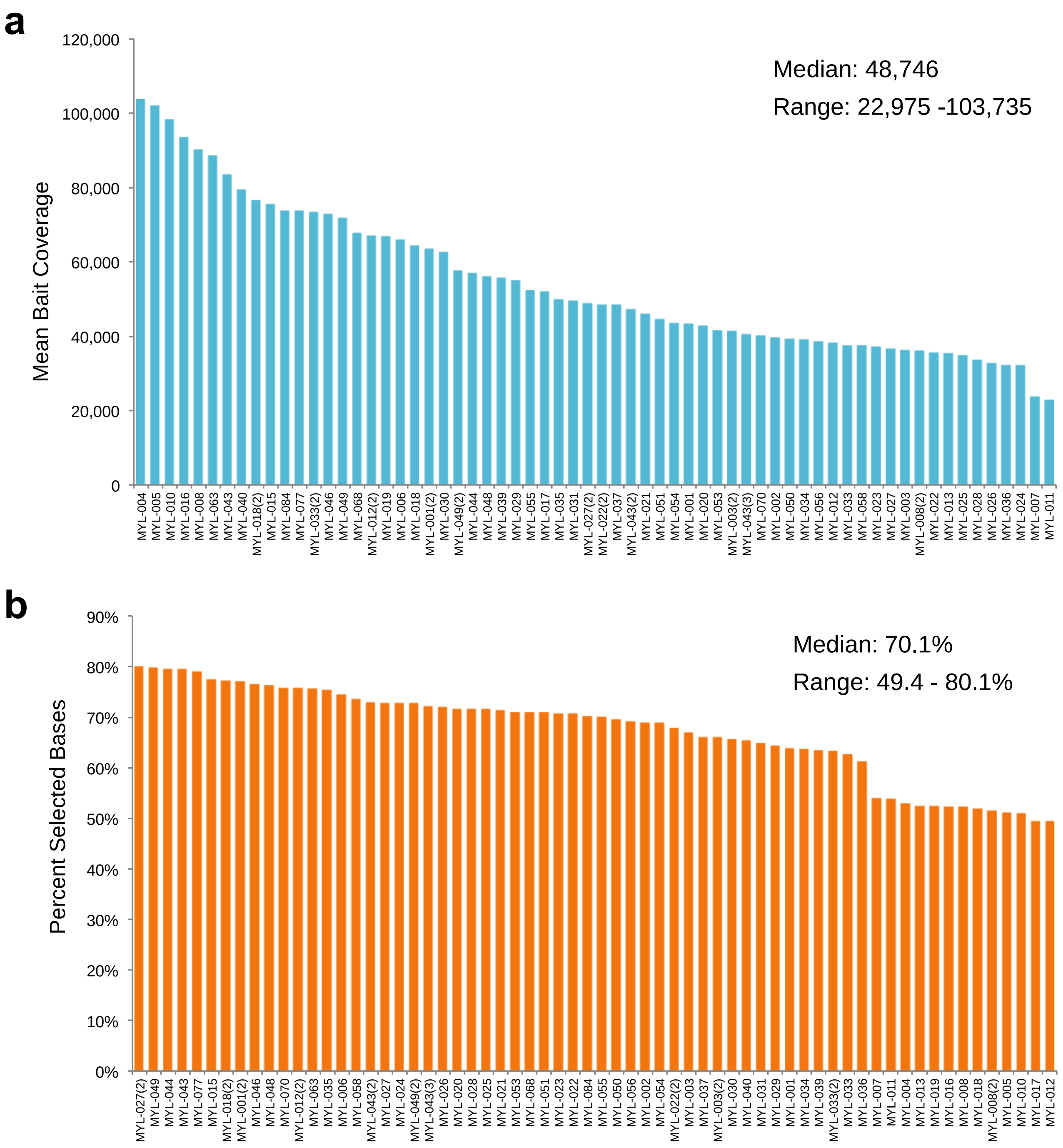

Supplementary Figure 5. Target DNA enrichment and mean bait coverage in cfDNA sequencing data. For 64cfDNA samples, after initial data processing following Genome Analysis Toolkit Best Practices[1], the mean bait coverage was evaluate using GATK Depth of Coverage analysis, counting the number of fragments instead of the number of reads (**a**). Percent selected bases (**b**) for each sample was determined as sum of on-target and near-target reads divided by total reads for each sample using the Picard[2] CalculateHsMetrics tool.

a LB-Seq sequencing data analysis pipeline

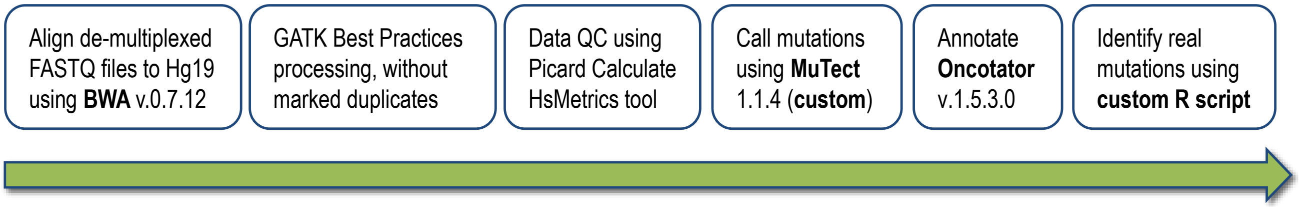

b

```
module load mutect/1.1.4
module load igenome-human/hg19
module load java/6

java -jar $mutect_dir/muTect-1.1.4.jar \
              --analysis_type MuTect \
              --reference_sequence $REF \
              --input_file:tumor /input/directory/file_name.bam \
              --out /output/directory/file_name.bam.call_stats \
              --intervals /directory/containing/target/panel/bed/file_name.bed \
              --tumor_f_pretest 0.0005f \
              --enable_extended_output \
              --downsampling_type NONE \
              --force_alleles \
              --gap_events_threshold 1000 \
              --fraction_contamination 0.00f \
              --coverage_file /output/directory/file_name.bam.coverage.wig.txt
```

c

```
module load oncotator/1.5.3.0

oncotator     --input_format=MAFLITE \
              --output_format=TCGAMAF \
              --db-dir $DB_DIR \
              --verbose /input/directory/call_stats/files/file.bam.call_stats \
              /directory/for/Oncotator/output/file_name.bam.call_stats.maf \
              hg19
```

d

```
module load R/3.2.2

Rscript       ctDNA_data_filtering_NEW.R \
              /path/to/directory/containing/Oncotator/output/maf/files/ \
              PROJECTNAME \
```

Supplementary Figure 6. LB-Seq sequencing data analysis pipeline. Flowchart for cfDNA data processing and analysis (**a**) and scripts used for running muTect (**b**), Oncotator (**c**), or Rscript filtering (**d**) analyses.

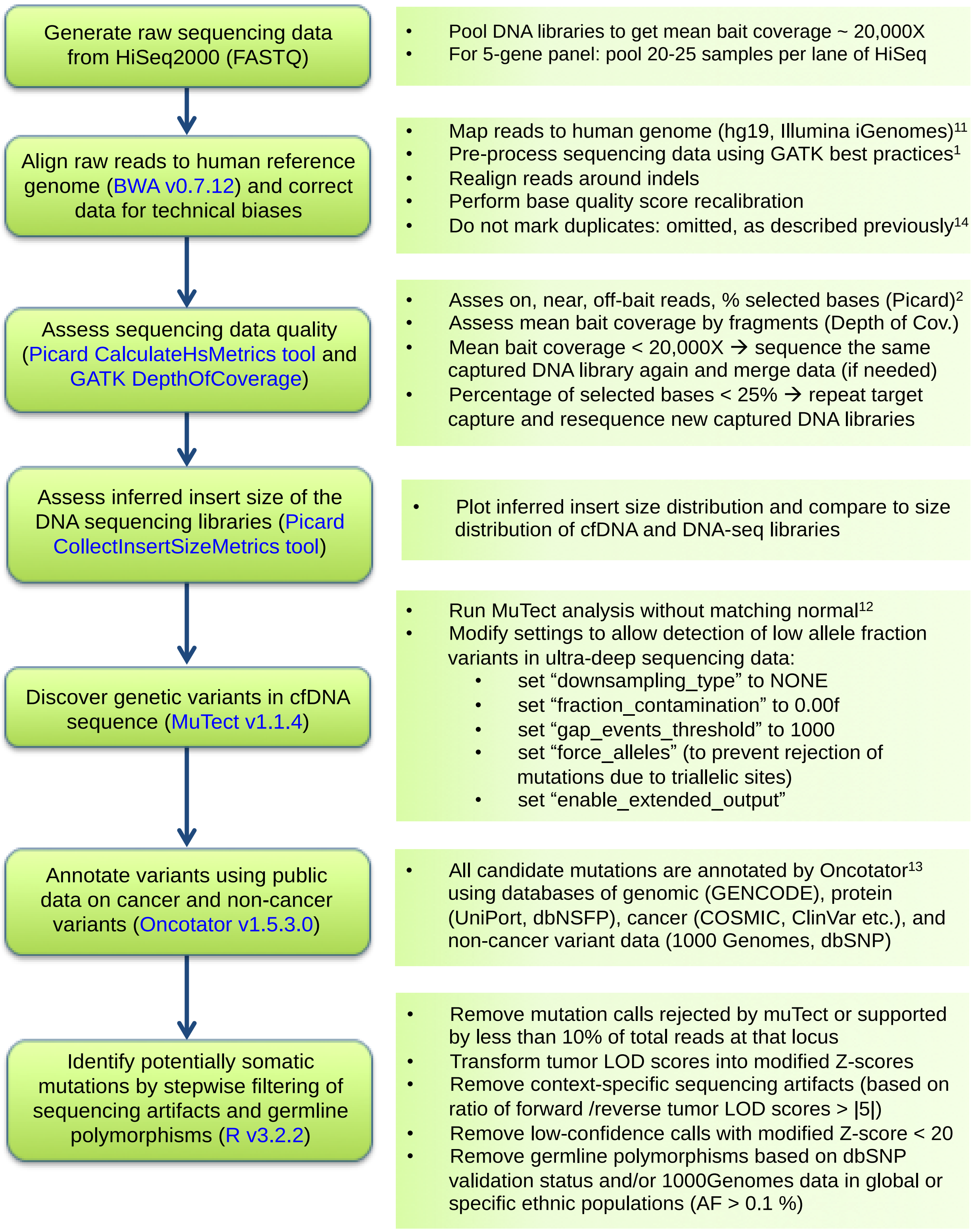

Supplementary Figure 7. Details of cfDNA sequencing data processing and filtering algorithm

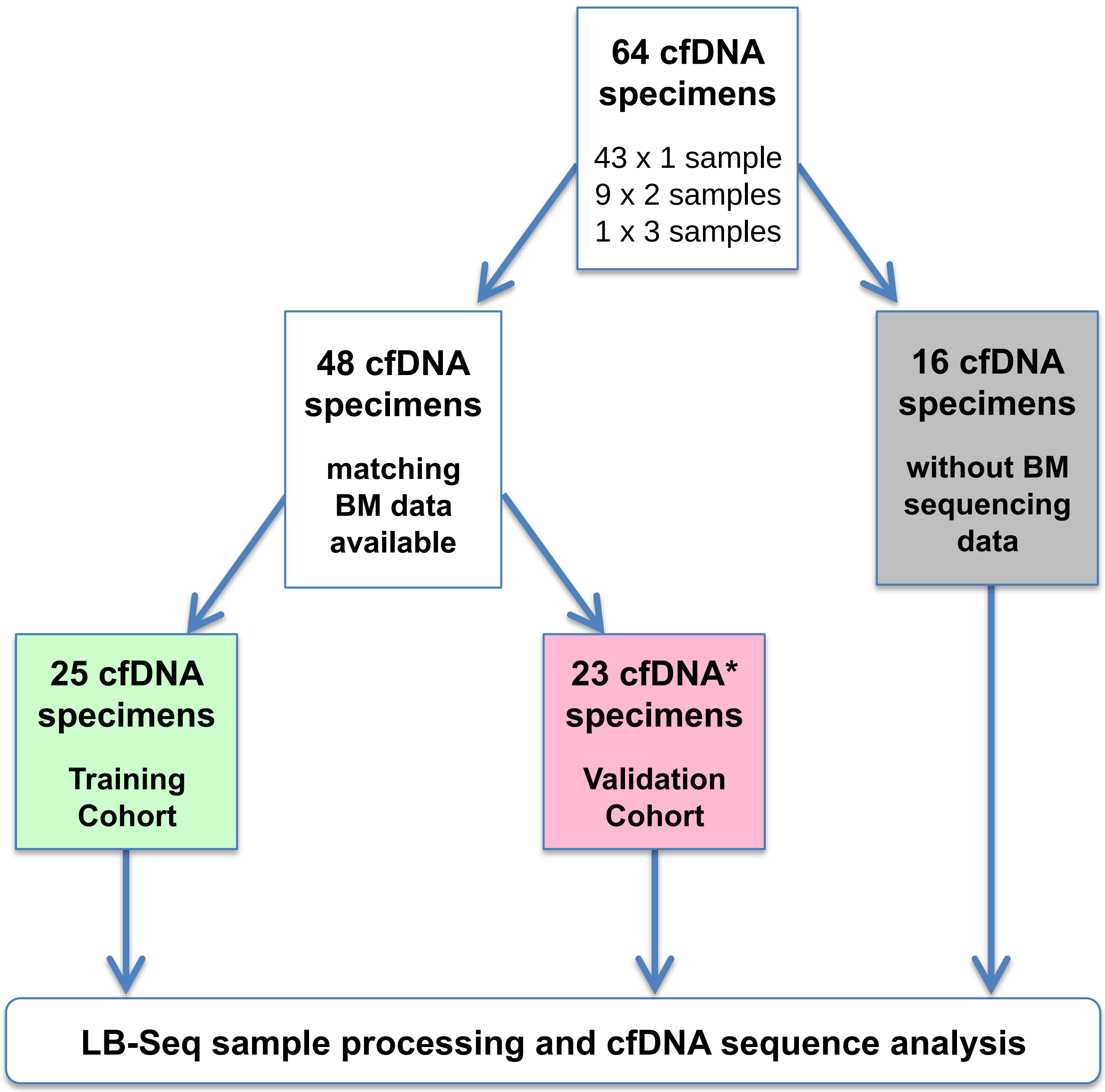

Supplementary Figure 8. Flowchart describing cfDNA specimen allocation within analysis cohorts. All cfDNA samples were sequenced to >20,000X mean coverage using LB-Seq method described in Supplementary Methods section. For matching BM-derived DNA samples in training and validation cohorts, the sequencing data were obtained from the clinical laboratory, CoMMpass collaboration, and/or generated in our laboratory using 5-gene panel targeted deep sequencing (>5,000X mean coverage). *Validation cohort included 4 serial cfDNA specimens from patients included in Training cohort that were excluded when calculating the sensitivity (concordance between LB-Seq and BM profiling data) and specificity of mutation calling for the validation cohort.

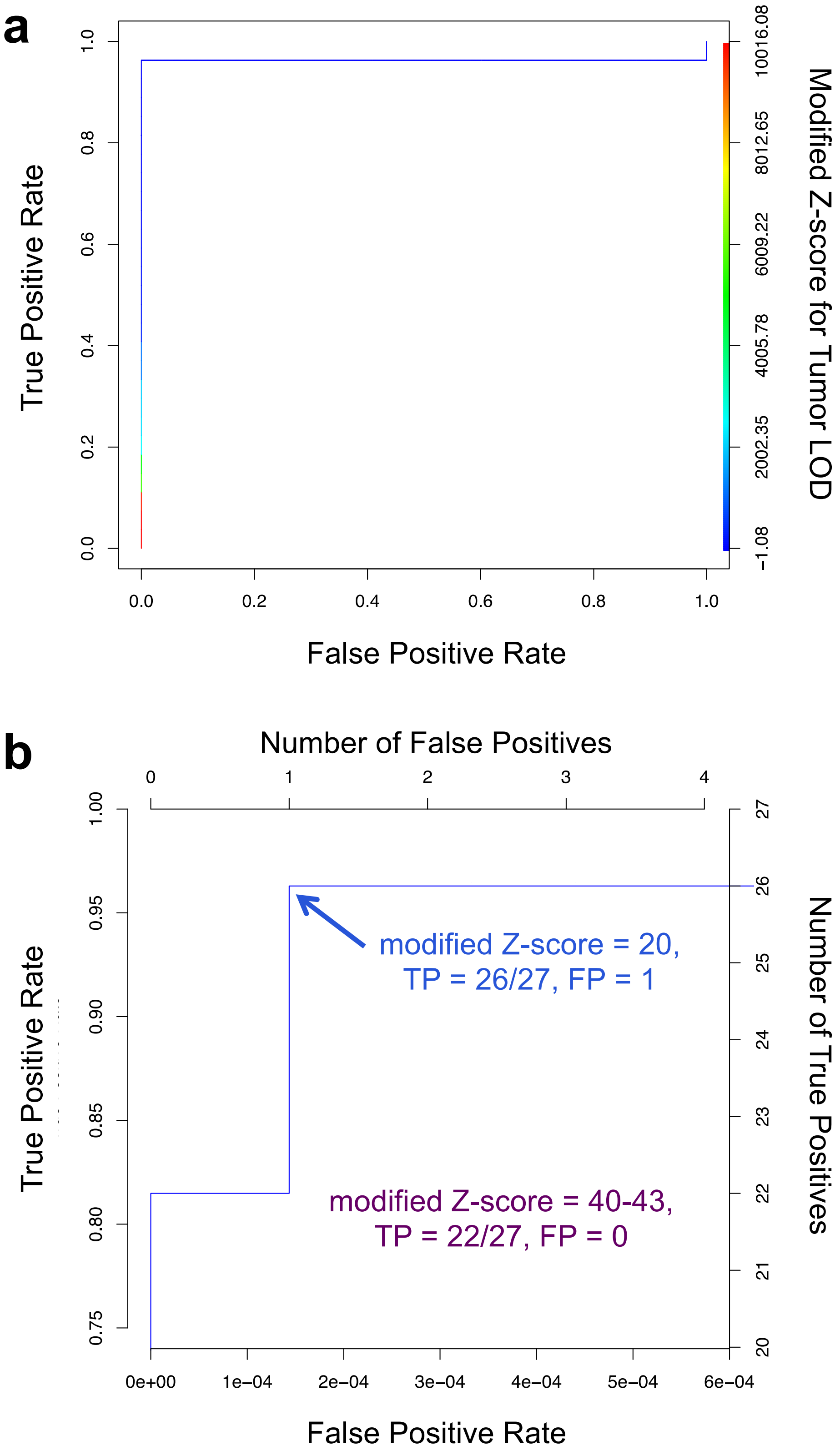
a
True Positive Rate
False Positive Rate
Modified Z-score for Tumor LOD
0.0
0.2
0.4
0.6
0.8
1.0
10016.08
8012.65
6009.22
4005.78
2002.35
−1.08
b
Number of False Positives
True Positive Rate
Number of True Positives
False Positive Rate
modified Z-score = 20,
TP = 26/27, FP = 1
modified Z-score = 40-43,
TP = 22/27, FP = 0
0e+00
1e−04
2e−04
3e−04
4e−04
5e−04
6e−04
0.75
0.80
0.85
0.90
0.95
1.00
20
21
22
23
24
25
26
27
0
1
2
3
4

Supplementary Figure 9. Receiver-operator curve (ROC) for identifying the threshold for calling likely somatic mutations. Training dataset consisting of 25 matched cfDNA and BM-derived DNA sample pairs was used to select a threshold for calling likely somatic mutations in cfDNA sequencing data. The aggregate muTect LOD scores for each mutation call generated by muTect were transformed into modified Z-scores, i.e., the number of median absolute deviations (MADs) from the median LOD score within each sample prior to analysis. The data for 25 cfDNA samples were then combined into one dataset used to generate the ROC curve for the range of the modified Z-score values used as thresholds and the corresponding True Positive and False Positive rates (**a**). All data were filtered to remove muTect-rejected mutations (prior to modified-Z-score calculations), context-specific sequencing artifacts, and known germline polymorphisms, as described in **Supplementary Figs 6** and **7**. True mutation calls we identified as those detected in the matching BM sample and were used to estimate the sensitivity (concordance between cfDNA and BM tumour DNA profiling data). Panel (**b**) shows the same ROC curve for 75-100% sensitivity and 0-4 false positive calls, which was used to select the modified Z-score threshold value for data filtering. When using modified Z-score value of 20 as a threshold, 26 of 27 true positive calls are detected in cfDNA (96.3% sensitivity) and the only apparent “false positive” mutation call (*PIK3CA* p.Y207* mutation in MYL-001) is believed to be a real mutation identified only in cfDNA (at AF = 0.28%) but not represented in the corresponding single-site BM aspirate. By increasing modified Z-score threshold to 40-43, this apparent “false positive” call can be eliminated; however, only 22 of 27 true positives will be identified (sensitivity of 81.5%). Hence, the modified Z-score value of 20 was selected as the optimal threshold value to maximize sensitivity and specificity of the LB-Seq method.

		Tumour Data		
		Pos	Neg	
cfDNA Data	Pos	True Positive	False Positive	PPV
	Neg	False Negative	True Negative	NPV
		SN	SP	

Training Cohort

		Tumour Data		
		26	104	
cfDNA Data	26	**25**	**1**	**96%**
	104	**1**	**103**	**99%**
		**96%**	**99%**	

Validation Cohort

		Tumour Data		
		20	77	
cfDNA Data	22	**19**	**3**	**86%**
	75	**1**	**74**	**99%**
		**95%**	**96%**	

Serial Samples from Training Cohort (n=4)*

		Tumour Data		
		5	14	
ctDNA Data	5	**5**	**0**	**100%**
	14	**0**	**14**	**100%**
		**100%**	**100%**	

		Tumour Data		
		51	195	
cfDNA Data	53	**49**	**4**	**92%**
	193	**2**	**191**	**99%**
		**96%**	**98%**	

Supplementary Figure 10. Concordance between cfDNA and BM tumour DNA sequencing data in training and validation cohorts. The training cohort consisted of 25 cfDNA samples from 23 MM patients with 25 matching BM-derived tumour DNA samples collected within 1 week of blood draw. The validation cohort included 19 matched cfDNA and BM-derived tumour DNA samples from 17 patients. We also analyzed four serial cfDNA samples (*) from patients in the training cohort that were excluded from validation of the Z-score threshold but were included in the overall calculations of sensitivity (concordance between cfDNA sequencing and BM tumour DNA profiling data) and specificity of mutation calling across all samples analyzed in this study (bottom diagram).

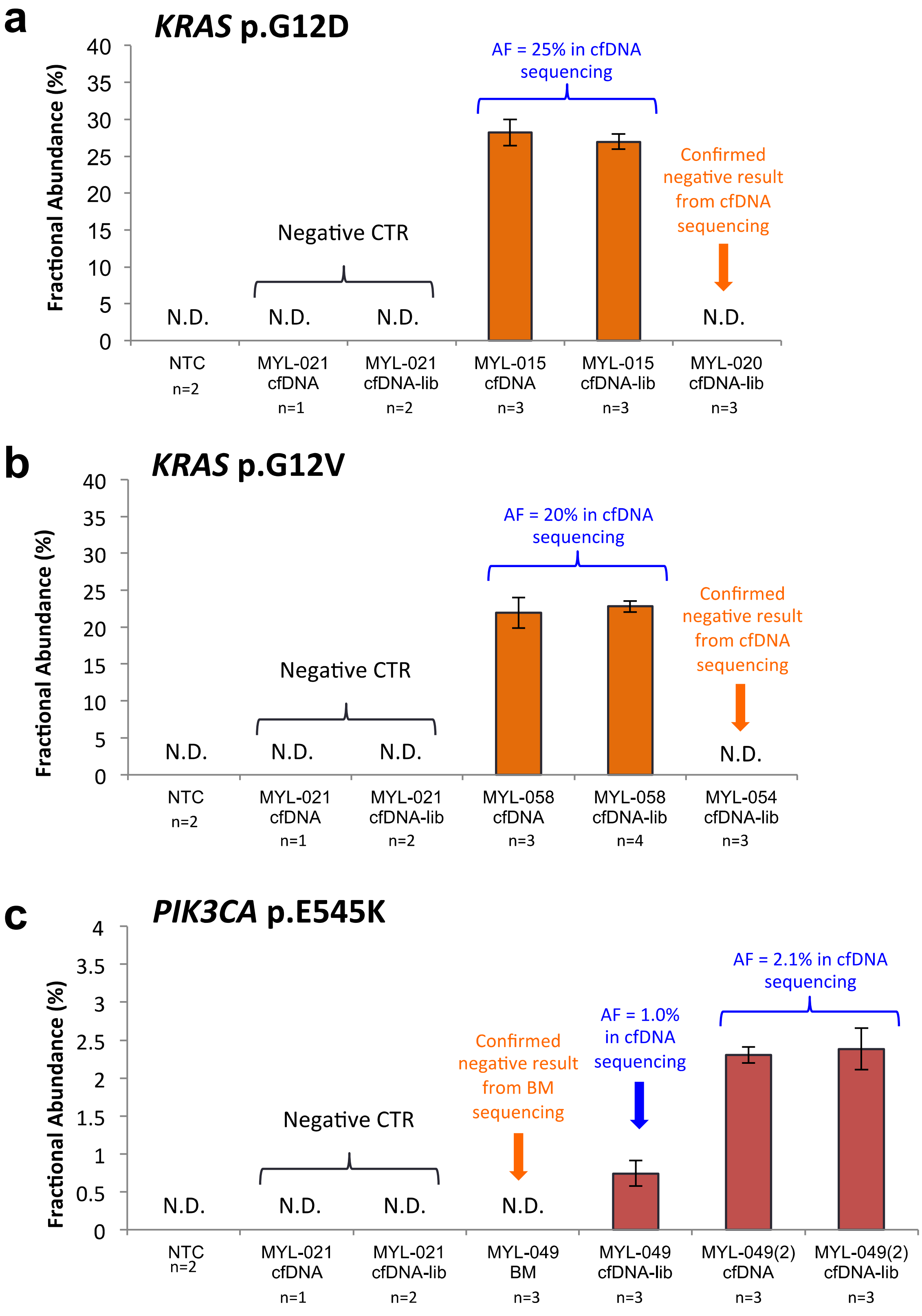
a
KRAS p.G12D
Fractional Abundance (%)
AF = 25% in cfDNA sequencing
Confirmed negative result from cfDNA sequencing
Negative CTR
N.D.
N.D.
N.D.
N.D.
NTC n=2
MYL-021 cfDNA n=1
MYL-021 cfDNA-lib n=2
MYL-015 cfDNA n=3
MYL-015 cfDNA-lib n=3
MYL-020 cfDNA-lib n=3
b
KRAS p.G12V
Fractional Abundance (%)
AF = 20% in cfDNA sequencing
Confirmed negative result from cfDNA sequencing
Negative CTR
N.D.
N.D.
N.D.
N.D.
NTC n=2
MYL-021 cfDNA n=1
MYL-021 cfDNA-lib n=2
MYL-058 cfDNA n=3
MYL-058 cfDNA-lib n=4
MYL-054 cfDNA-lib n=3
c
PIK3CA p.E545K
Fractional Abundance (%)
AF = 2.1% in cfDNA sequencing
AF = 1.0% in cfDNA sequencing
Confirmed negative result from BM sequencing
Negative CTR
N.D.
N.D.
N.D.
N.D.
NTC n=2
MYL-021 cfDNA n=1
MYL-021 cfDNA-lib n=2
MYL-049 BM n=3
MYL-049 cfDNA-lib n=3
MYL-049(2) cfDNA n=3
MYL-049(2) cfDNA-lib n=3

Supplementary Figure 11. Validation of discordant mutation calls from LB-Seq analysis by ddPCR. Droplet digital PCR (ddPCR) analysis was used to validate three discordant calls present in the training and validation datasets: *KRAS* p.G12D mutation undetectable in MYL-020-cfDNA by LB-Seq (**a**); *KRAS* p.G12V mutation undetectable in MYL-054-cfDNA by LB-Seq (**b**); and *PIK3CA* p.E545K mutation undetectable in MYL-049-BM (**c**) but detected in two cfDNA samples from this patient. Validated ddPCR assays for each mutations of interest were obtained from Bio-Rad Canada (Mississauga, Ontario). We used 25-50 ηg of extracted DNA (MYL-049-BM, MYL-049(2)-cfDNA) or the corresponding adapter-ligated DNA library (MYL-020-cfDNA-lib, MYL-054-cfDNA-lib, MYL-049-cfDNA-lib, and MYL-049(2)-cfDNA-lib) for each ddPCR reaction. Each assay had a different positive control: MYL-015-cfDNA for *KRAS* p.G12D (**a**), MYL-058-cfDNA for *KRAS* p.G12V (**b**), and MYL-049-cfDNA or MYL-049(2)-cfDNA for *PIK3CA* p.E545K (**c**). The same negative control sample (MYL-021 cfDNA) and no-template control (NTC, water) were used in all three assays. Bars and error bars represent mean ± s.d. for the number of technical replicates specified in the figure (shown underneath sample IDs). N.D., not detected by ddPCR analysis.

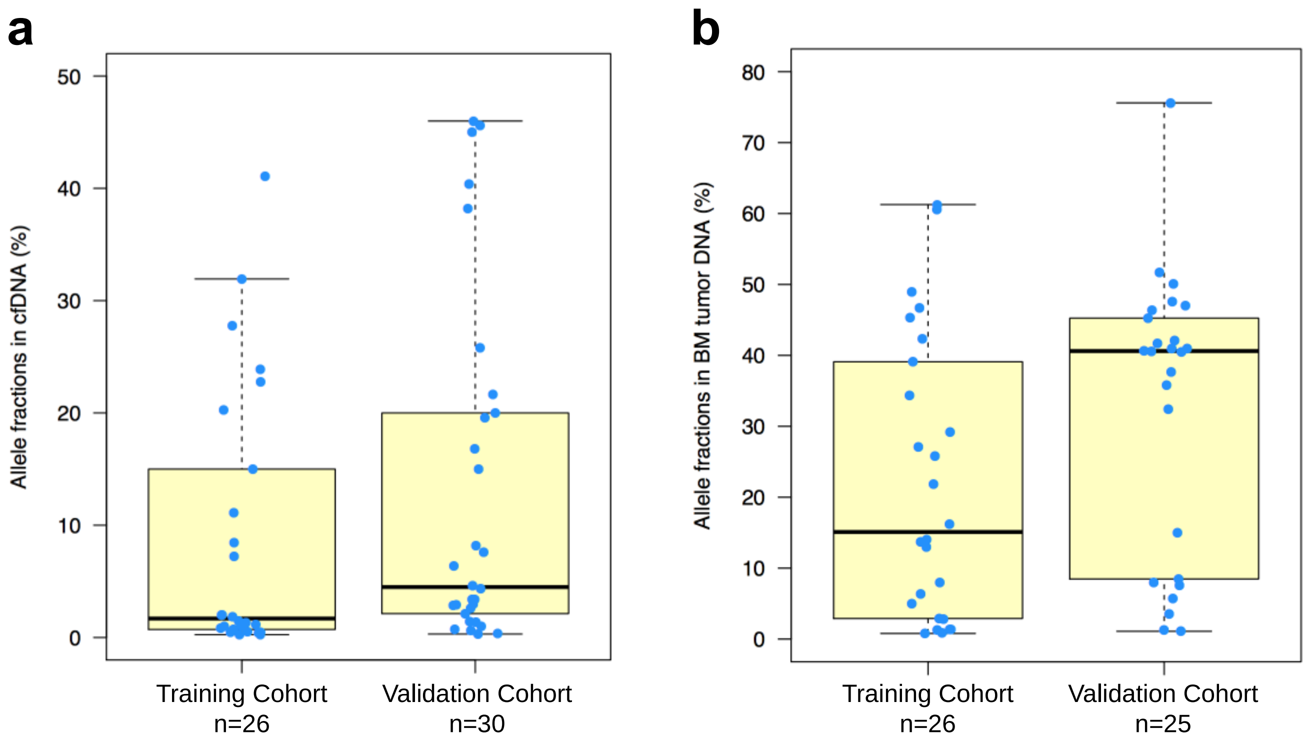

Supplementary Figure 12. Comparison of mutant allele frequencies in training and validation cohorts. The distributions of mutant allele frequencies detected in cfDNA (**a**) or BM tumour DNA (**b**) sequencing data in training and validation cohort samples are shown as box plots, where the central rectangle spans the first to the third quartile (interquartile range or IQR). A segment inside the rectangle shows the median, and "whiskers" above and below the box show the value 1.5×IQR above or below the third or the first quartile, respectively. The number of mutations included in each analysis is shown under each group name in the figure. Wilcoxon signed-rank test was used for comparison of two groups in each panel with *P* value of 0.05 considered statistically significant.

Cohort	BM Analysis			Sample ID	*KRAS*		*NRAS*		*BRAF*		*EGFR*		*PIK3CA*	
	Clinical Lab	CoMMpass	5-gene panel		Protein Change	ctDNA allele fraction	Protein Change	ctDNA allele fraction	Protein Change	ctDNA allele fraction	Protein Change	ctDNA allele fraction	Protein Change	ctDNA allele fraction
Without BM Data				MYL-006	p.G12D	6.4%								
				MYL-018(2)	p.A146T	6.3%								
				MYL-029	p.A59L†	2.0%								
	F			MYL-040	p.A146V	1.8%								
				MYL-043(2)	p.G13D	7.6%								
				MYL-015	p.G12D	25%							p.D64N	6.3%
				MYL-013			p.Q61L	2.7%						
				MYL-025			p.Q61K	2.5%						
				MYL-021			p.G12A	0.29%						
	F			MYL-024					p.V600E	0.75%				
				MYL-008										
				MYL-008(2)										
				MYL-010										
				MYL-011										
	F			MYL-035										
	F			MYL-048										

Supplementary Figure 13. Likely somatic mutations identified by LB-Seq in cfDNA samples without BM-derived tumour DNA sequencing data. Sample IDs followed by (2) represent serial samples obtained on a separate clinical visit. †Single amino acid substitution resulting from a two-base genomic substitution; F, BM sample was collected but failed clinical profiling due to insufficient yield of malignant cells or poor DNA quality.

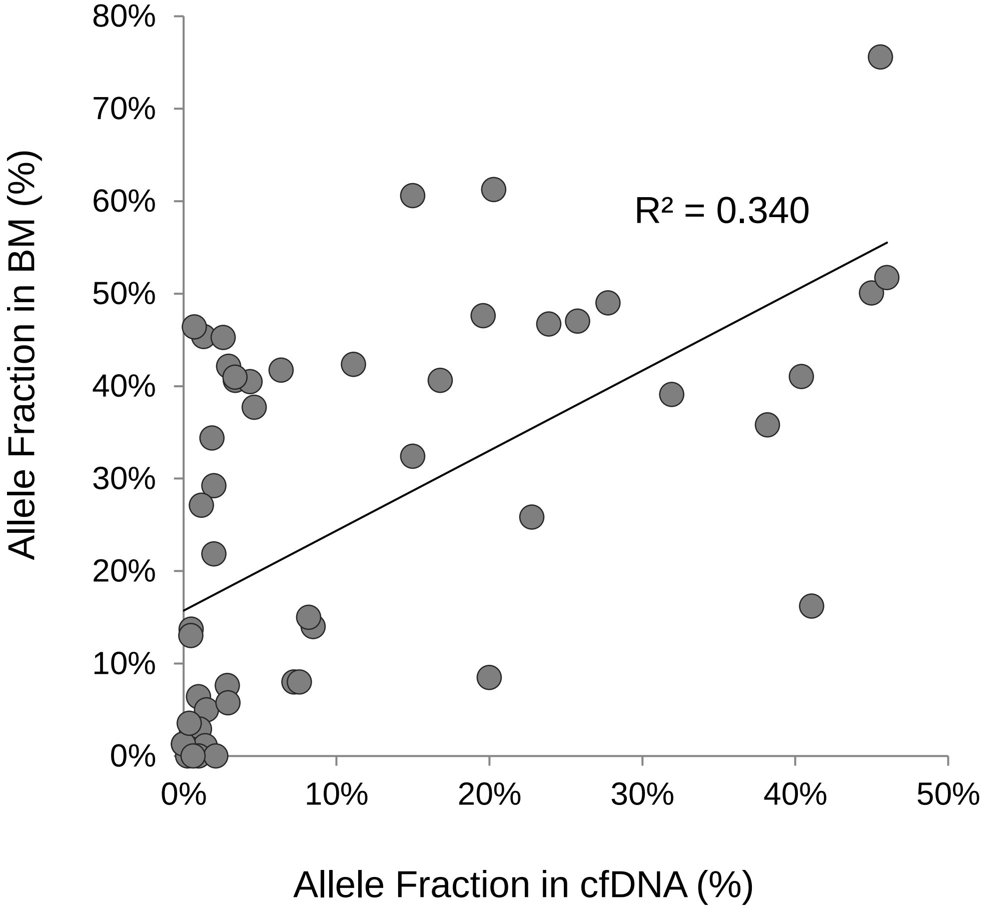

Supplementary Figure 14. Comparison of mutant allele frequencies in cfDNA and BM-derived tumour DNA in all samples. For 48 cfDNA samples with matching BM-derived tumour DNA sequencing data available, a scatter plot of corresponding cfDNA and BM-derived DNA allele fractions was generated. Linear regression was then assessed to determine the strength of correlation (R^2 value). The observed lack of correlation between cfDNA and BM allele fractions is likely due to the differences in the proportion of tumour DNA present in the total cfDNA extracted from plasma of each patient. Notably, for patients with multiple mutations detected in cfDNA sequence analysis, much stronger correlation of cfDNA and BM allele fractions was detected, as shown in **Figure 5** of the main text.

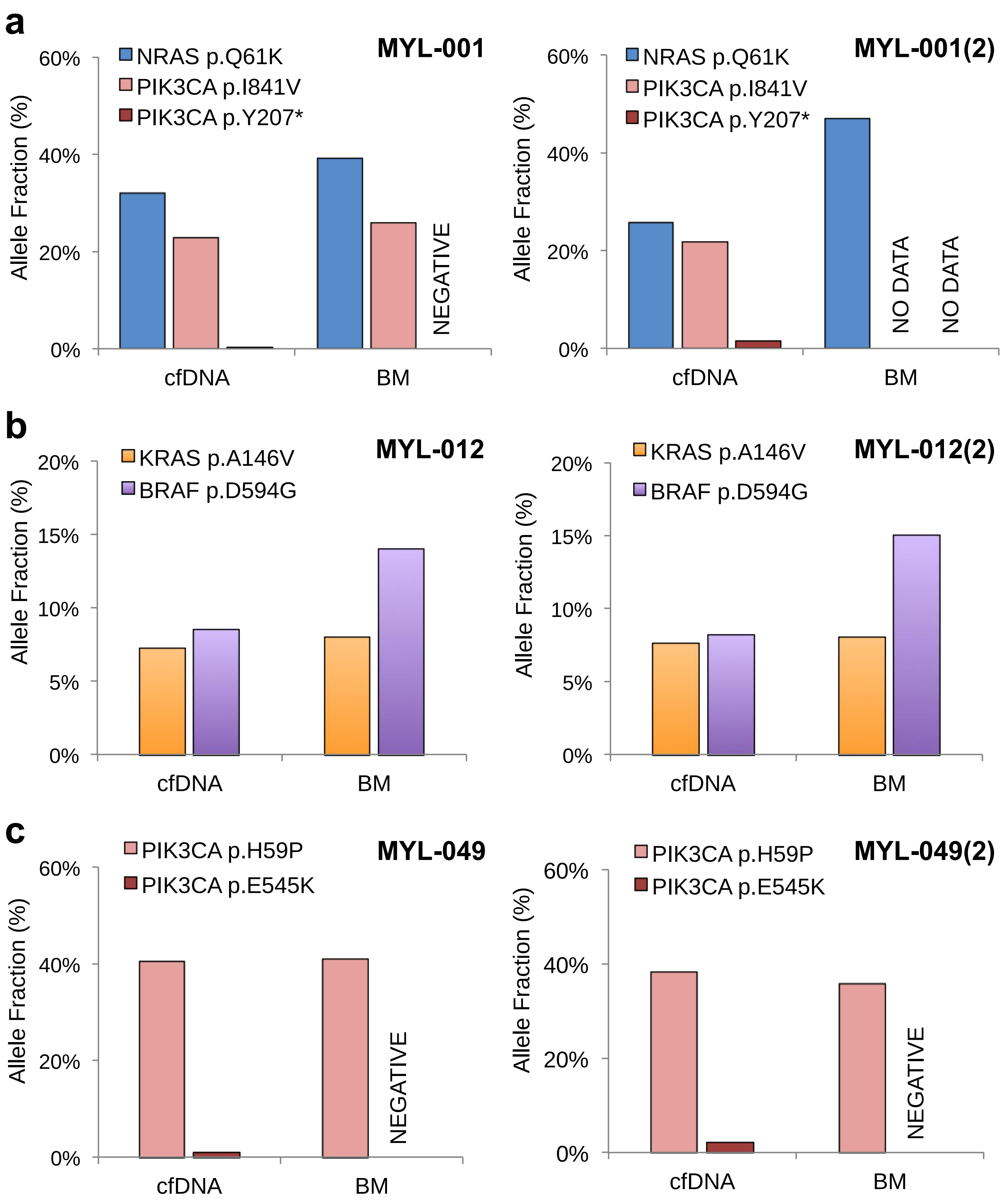
a
MYL-001
NRAS p.Q61K
PIK3CA p.I841V
PIK3CA p.Y207*
Allele Fraction (%)
60%
40%
20%
0%
NEGATIVE
cfDNA
BM
MYL-001(2)
NRAS p.Q61K
PIK3CA p.I841V
PIK3CA p.Y207*
NO DATA
NO DATA
b
MYL-012
KRAS p.A146V
BRAF p.D594G
20%
15%
10%
5%
0%
MYL-012(2)
c
MYL-049
PIK3CA p.H59P
PIK3CA p.E545K
NEGATIVE
MYL-049(2)

Supplementary Figure 15. Clonal hierarchies in serial cfDNA and matching BM DNA samples with multiple mutations. This figure complements data in **Figure 5** of the main text. Each panel compares the allele fractions for all mutations detected in two serial cfDNA samples and two matching BM-derived tumour DNA samples from MYL-001 (**a**), MYL-012 (**b**), and MYL-049 (**c**) patients. “Negative” indicates that the corresponding mutation was not detected in the BM samples. “No Data” indicates that the corresponding mutation was not examined in the BM sample because a different target panel was used for BM sequencing which did not include this genomic locus.

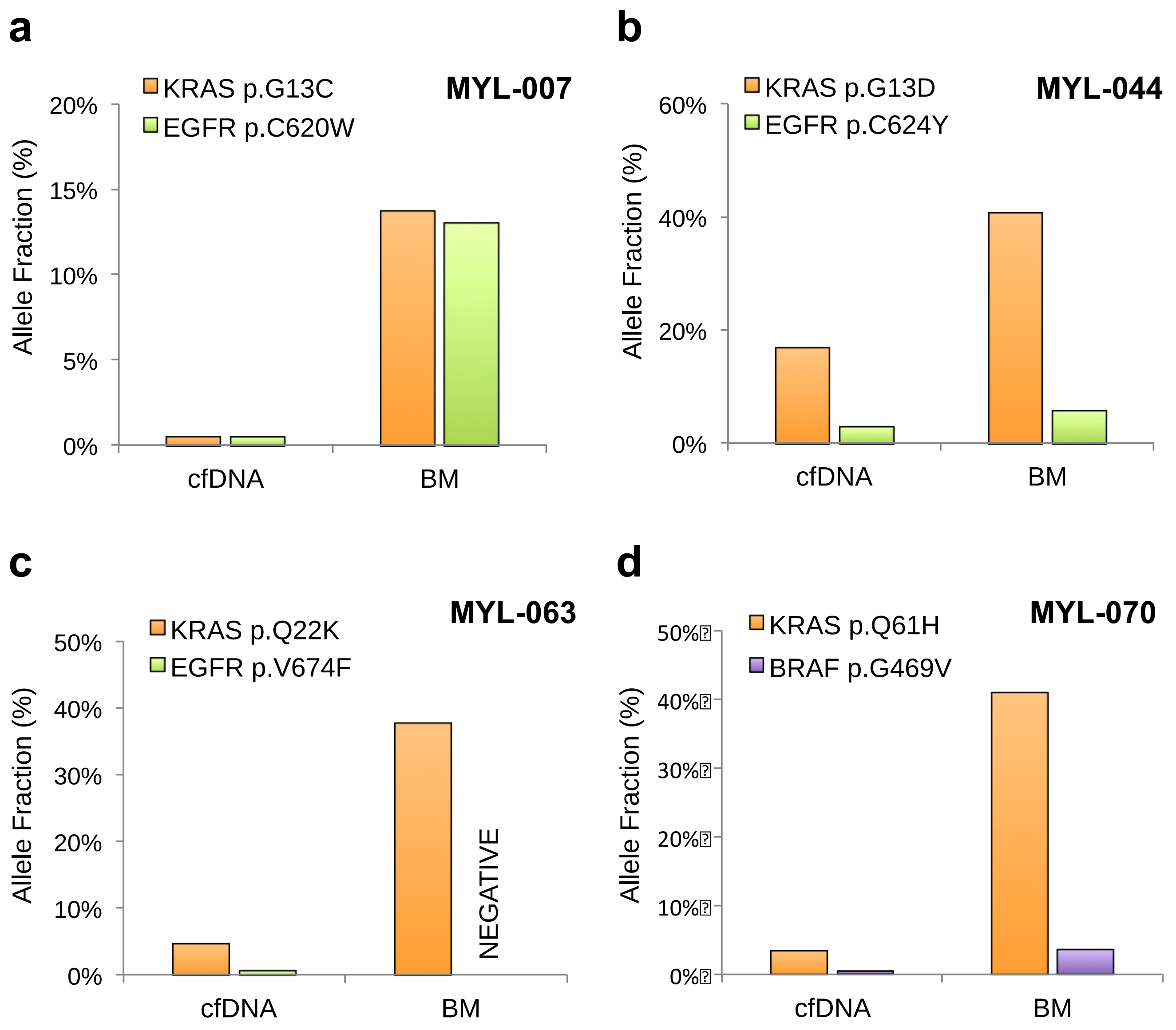

Supplementary Figure 16. Clonal hierarchies detected in other cfDNA and BM tumour DNA samples with multiple mutations. This figure complements data in **Figure 5** of the main text and **Supplementary Figure 15**. Each panel compares the allele fractions for all mutations detected in matching cfDNA and BM-derived tumour DNA samples from MYL-007 (**a**), MYL-044 (**b**), MYL-063 (**c**), and MYL-070 (**d**).

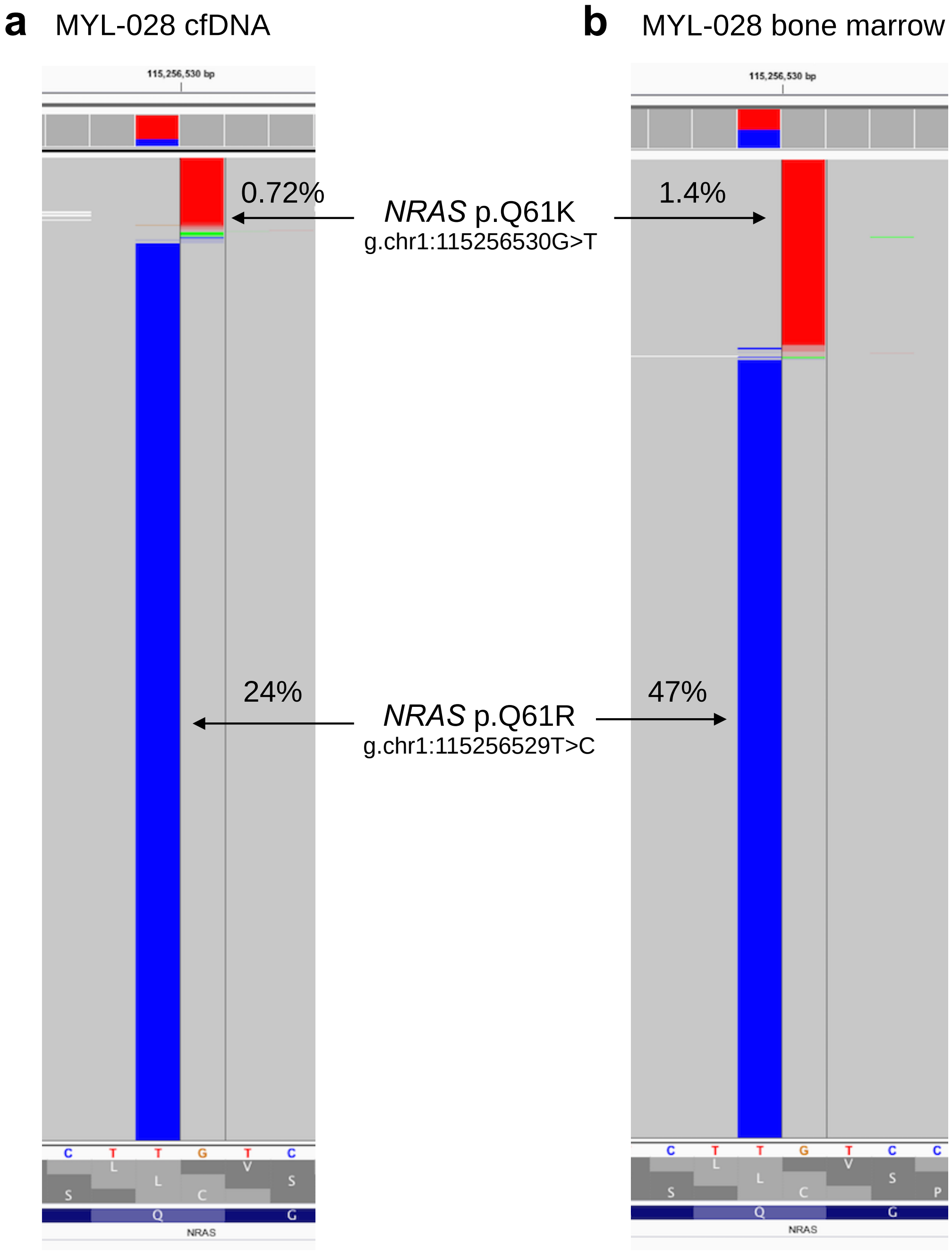

Supplementary Figure 17. ***NRAS*** **p.Q61R and p.Q61K mutations in MYL-028 cfDNA and matching BM tumour DNA sequencing data.** Deep sequencing data for MYL-028 cfDNA (**a**) and BM-derived DNA (**b**) were generated using 5-gene panel targeted sequencing and visualized using the Integrated Genome Viewer software (“squished” view). Reads were sorted by base at chr1:115,256,529 locus and then again sorted by base at chr1:115,256,530 locus. Red bars show G>T substitutions at the chr1:115,256,530 locus. Blue bars show T>C substitution at the chr1:115,256,529 locus.

Supplementary Table 1. Comparison of BM sequencing data from different sources

Sample ID	Gene, protein change	Tumour allele fractions in the BM-derived DNA		
		Clinical laboratory	CoMMpass collaboration	Sequenced using 5-gene panel
MYL-001	*NRAS*, p.Q61K	47%		39%
	PIK3CA, p.I841V	Not covered by the panel		26%
MYL-001(2)	*NRAS*, p.Q61K	47%		
	PIK3CA, p.I841V	Not covered by the panel		
MYL-002	*NRAS*, p.G13D	No mutations		0.93%
MYL-003	*NRAS*, p.Q61K	50%		46%
MYL-003(2)	*NRAS*, p.Q61K	48%		61%
MYL-004		No mutations		No mutations
MYL-018	*KRAS*, p.A146T	20%		22%
MYL-019	*NRAS*, p.G13R	59%		60%
MYL-020	*KRAS*, p.G12V	10%		4.6%
	KRAS, p.Q61H	Not detected		2.6%
	KRAS, p.G12D	Not detected		2.0%
MYL-022	*NRAS*, p.Q61R	22%		26%
MYL-023	*KRAS*, p.Q61H	5%		
	KRAS, p.G13C	N.D. 1.4%*		
	BRAF, p.V600E	N.D. 2.9%*		
	EGFR, p.A859S	N.D. 0.8%*		
MYL-026	*KRAS*, p.Q61H	36%		29%
MYL-027	*EGFR* p.E551K	Not covered by the panel		14%
MYL-028	*NRAS*, p.Q61R	39%		47%
	NRAS, p.Q61K	Not detected		1.3%
MYL-030	*KRAS*, p.G13D	41%		43%
MYL-034		No mutations		No mutations
MYL-036		No mutations		No mutations
MYL-037		No mutations		No mutations
MYL-039	*NRAS*, p.Q61K	31%		34%
MYL-043	*KRAS*, p.G13D	9%		8.6%

* mutations that were initially not called by the AMDL clinical laboratory; raw sequencing data was reanalyzed using the same filtering algorithm as that used for 5-gene panel analysis and the allele fractions shown (<5%) were detected.

Supplementary Table 2. Types of primary malignancies used for comparison of cfDNA circulating levels to MM

Malignancy type	Number of samples
Prostate	12
Colorectal	10
Breast	7
Lung	5
Gynaecological	5
Pancreatobiliary	4
Endometrial	2
GE junction	1
Sarcoma	1
Thyroid	1
Primary unknown	8

Supplementary Table 3. Biotinylated ssDNA capture probes included in the IDT xGen Lockdown Custom Probes Mini Pool.

Probe genomic location*				Target genomic region	
Chr	Start	End	Strand	Gene and exon	Genomic coordinates
1	115251096	115251215	-	*NRAS* exon 5	chr1:115251096-115251335
1	115251216	115251335	-	*NRAS* exon 5	chr1:115251096-115251335
1	115252150	115252269	-	*NRAS* exon 4	chr1:115252150-115252389
1	115252270	115252389	-	*NRAS* exon 4	chr1:115252150-115252389
1	115256391	115256510	-	*NRAS* exon 3	chr1:115256391-115256630
1	115256511	115256630	-	*NRAS* exon 3	chr1:115256391-115256630
1	115258607	115258726	-	*NRAS* exon 2	chr1:115258607-115258846
1	115258727	115258846	-	*NRAS* exon 2	chr1:115258607-115258846
3	178916550	178916669	+	*PIK3CA* exon 2	chr3:178916550-178917029
3	178916670	178916789	+	*PIK3CA* exon 2	chr3:178916550-178917029
3	178916790	178916909	+	*PIK3CA* exon 2	chr3:178916550-178917029
3	178916910	178917029	+	*PIK3CA* exon 2	chr3:178916550-178917029
3	178917463	178917582	+	*PIK3CA* exon 3	chr3:178917463-178917702
3	178917583	178917702	+	*PIK3CA* exon 3	chr3:178917463-178917702
3	178919024	178919143	+	*PIK3CA* exon 4	chr3:178919024-178919383
3	178919134	178919253	+	*PIK3CA* exon 4	chr3:178919024-178919383
3	178919244	178919363	+	*PIK3CA* exon 4	chr3:178919024-178919383
3	178921275	178921394	+	*PIK3CA* exon 5	chr3:178921275-178921634
3	178921395	178921514	+	*PIK3CA* exon 5	chr3:178921275-178921634
3	178921515	178921634	+	*PIK3CA* exon 5	chr3:178921275-178921634
3	178922274	178922393	+	*PIK3CA* exon 6	chr3:178922274-178922393
3	178927316	178927435	+	*PIK3CA* exon 7	chr3:178927316-178927555
3	178927436	178927555	+	*PIK3CA* exon 7	chr3:178927316-178927555
3	178927931	178928050	+	*PIK3CA* exon 8,9	chr3:178927931-178928406
3	178928050	178928169	+	*PIK3CA* exon 8,9	chr3:178927931-178928406
3	178928168	178928287	+	*PIK3CA* exon 8,9	chr3:178927931-178928406
3	178928287	178928406	+	*PIK3CA* exon 8,9	chr3:178927931-178928406
3	178935941	178936060	+	*PIK3CA* exon 10	chr3:178935941-178936180
3	178936061	178936180	+	*PIK3CA* exon 10	chr3:178935941-178936180
3	178936965	178937084	+	*PIK3CA* exon 11	chr3:178936965-178937084
3	178937322	178937441	+	*PIK3CA* exon 12	chr3:178937322-178937561
3	178937424	178937543	+	*PIK3CA* exon 12	chr3:178937322-178937561
3	178937669	178937788	+	*PIK3CA* exon 13	chr3:178937669-178937908
3	178937789	178937908	+	*PIK3CA* exon 13	chr3:178937669-178937908
3	178938740	178938859	+	*PIK3CA* exon 14	chr3:178938740-178938979
3	178938860	178938979	+	*PIK3CA* exon 14	chr3:178938740-178938979
3	178941803	178941922	+	*PIK3CA* exon 15	chr3:178941803-178942042

Probe genomic location*				Target genomic region	
Chr	Start	End	Strand	Gene and exon	Genomic coordinates
3	178941923	178942042	+	*PIK3CA* exon 15	chr3:178941803-178942042
3	178942429	178942548	+	*PIK3CA* exon 16	chr3:178942429-178942668
3	178942549	178942668	+	*PIK3CA* exon 16	chr3:178942429-178942668
3	178943730	178943849	+	*PIK3CA* exon 17	chr3:178943730-178943849
3	178947026	178947145	+	*PIK3CA* exon 18	chr3:178947026-178947265
3	178947146	178947265	+	*PIK3CA* exon 18	chr3:178947026-178947265
3	178947731	178947850	+	*PIK3CA* exon 19,20	chr3:178947731-178948208
3	178947850	178947969	+	*PIK3CA* exon 19,20	chr3:178947731-178948208
3	178947970	178948089	+	*PIK3CA* exon 19,20	chr3:178947731-178948208
3	178948089	178948208	+	*PIK3CA* exon 19,20	chr3:178947731-178948208
3	178951838	178951957	+	*PIK3CA* exon 21	chr3:178951838-178952197
3	178951958	178952077	+	*PIK3CA* exon 21	chr3:178951838-178952197
3	178952078	178952197	+	*PIK3CA* exon 21	chr3:178951838-178952197
7	55086955	55087073	+	*EGFR* exon 1	chr7:55086955-55087074
7	55209935	55210054	+	*EGFR* exon 2	chr7:55209935-55210174
7	55210055	55210174	+	*EGFR* exon 2	chr7:55209935-55210174
7	55210970	55211089	+	*EGFR* exon 3	chr7:55210970-55211209
7	55211090	55211209	+	*EGFR* exon 3	chr7:55210970-55211209
7	55214247	55214366	+	*EGFR* exon 4	chr7:55214247-55214486
7	55214367	55214486	+	*EGFR* exon 4	chr7:55214247-55214486
7	55218962	55219081	+	*EGFR* exon 5	chr7:55218962-55219081
7	55220179	55220298	+	*EGFR* exon 6	chr7:55220179-55220418
7	55220299	55220418	+	*EGFR* exon 6	chr7:55220179-55220418
7	55221655	55221774	+	*EGFR* exon 7	chr7:55221655-55221894
7	55221775	55221894	+	*EGFR* exon 7	chr7:55221655-55221894
7	55223462	55223581	+	*EGFR* exon 8	chr7:55223462-55223701
7	55223582	55223701	+	*EGFR* exon 8	chr7:55223462-55223701
7	55224170	55224289	+	*EGFR* exon 9	chr7:55224170-55224409
7	55224290	55224409	+	*EGFR* exon 9	chr7:55224170-55224409
7	55224429	55224548	+	*EGFR* exon 10	chr7:55224429-55224554
7	55224435	55224554	+	*EGFR* exon 10	chr7:55224429-55224554
7	55225342	55225461	+	*EGFR* exon 11	chr7:55225342-55225461
7	55227812	55227931	+	*EGFR* exon 12	chr7:55227812-55228051
7	55227932	55228051	+	*EGFR* exon 12	chr7:55227812-55228051
7	55229139	55229258	+	*EGFR* exon 13	chr7:55229139-55229378
7	55229259	55229378	+	*EGFR* exon 13	chr7:55229139-55229378
7	55231412	55231531	+	*EGFR* exon 14	chr7:55231412-55231531
7	55232932	55233051	+	*EGFR* exon 15	chr7:55232932-55233171
7	55233052	55233171	+	*EGFR* exon 15	chr7:55232932-55233171

Probe genomic location*				Target genomic region	
Chr	Start	End	Strand	Gene and exon	Genomic coordinates
7	55236160	55236279	+	*EGFR* exon 16	chr7:55236160-55236279
7	55237939	55238058	+	*EGFR* exon 16	chr7:55237939-55238298
7	55238059	55238178	+	*EGFR* exon 16	chr7:55237939-55238298
7	55238179	55238298	+	*EGFR* exon 16	chr7:55237939-55238298
7	55238828	55238947	+	*EGFR* exon 16	chr7:55238828-55238947
7	55240627	55240746	+	*EGFR* exon 17	chr7:55240627-55240866
7	55240747	55240866	+	*EGFR* exon 17	chr7:55240627-55240866
7	55241556	55241675	+	*EGFR* exon 18	chr7:55241556-55241795
7	55241676	55241795	+	*EGFR* exon 18	chr7:55241556-55241795
7	55242405	55242524	+	*EGFR* exon 19	chr7:55242405-55242524
7	55248959	55249078	+	*EGFR* exon 20	chr7:55248959-55249198
7	55249079	55249198	+	*EGFR* exon 20	chr7:55248959-55249198
7	55259370	55259489	+	*EGFR* exon 21	chr7:55259370-55259609
7	55259490	55259609	+	*EGFR* exon 21	chr7:55259370-55259609
7	55260437	55260556	+	*EGFR* exon 22	chr7:55260437-55260556
7	55266364	55266483	+	*EGFR* exon 23	chr7:55266364-55266603
7	55266484	55266603	+	*EGFR* exon 23	chr7:55266364-55266603
7	55267998	55268117	+	*EGFR* exon 24	chr7:55267998-55268117
7	55268845	55268964	+	*EGFR* exon 25	chr7:55268845-55269084
7	55268965	55269084	+	*EGFR* exon 25	chr7:55268845-55269084
7	55269392	55269511	+	*EGFR* exon 26	chr7:55269392-55269511
7	55270157	55270276	+	*EGFR* exon 27	chr7:55270145-55270384
7	55270265	55270384	+	*EGFR* exon 27	chr7:55270145-55270384
7	55272930	55273049	+	*EGFR* exon 28	chr7:55272890-55273369
7	55273037	55273156	+	*EGFR* exon 28	chr7:55272890-55273369
7	55273143	55273262	+	*EGFR* exon 28	chr7:55272890-55273369
7	55273250	55273369	+	*EGFR* exon 28	chr7:55272890-55273369
7	140434364	140434483	-	*BRAF* exon 18	chr7:140434364-140434603
7	140434474	140434593	-	*BRAF* exon 18	chr7:140434364-140434603
7	140439560	140439679	-	*BRAF* exon 17	chr7:140439560-140439799
7	140439680	140439799	-	*BRAF* exon 17	chr7:140439560-140439799
7	140449033	140449152	-	*BRAF* exon 16	chr7:140449033-140449272
7	140449153	140449272	-	*BRAF* exon 16	chr7:140449033-140449272
7	140453015	140453134	-	*BRAF* exon 15	chr7:140453015-140453254
7	140453135	140453254	-	*BRAF* exon 15	chr7:140453015-140453254
7	140453951	140454070	-	*BRAF* exon 14	chr7:140453951-140454070
7	140476681	140476800	-	*BRAF* exon 13	chr7:140476681-140476920
7	140476801	140476920	-	*BRAF* exon 13	chr7:140476681-140476920
7	140477774	140477893	-	*BRAF* exon 12	chr7:140477774-140477893

Probe genomic location*				Target genomic region	
**Chr**	**Start**	**End**	**Strand**	**Gene and exon**	**Genomic coordinates**
7	140481315	140481434	-	*BRAF* exon 11	chr7:140481315-140481554
7	140481435	140481554	-	*BRAF* exon 11	chr7:140481315-140481554
7	140482770	140482889	-	*BRAF* exon 10	chr7:140482770-140483009
7	140482890	140483009	-	*BRAF* exon 10	chr7:140482770-140483009
7	140487307	140487426	-	*BRAF* exon 9	chr7:140487307-140487426
7	140494068	140494187	-	*BRAF* exon 8	chr7:140494068-140494307
7	140494188	140494307	-	*BRAF* exon 8	chr7:140494068-140494307
7	140500102	140500221	-	*BRAF* exon 7	chr7:140500102-140500341
7	140500222	140500341	-	*BRAF* exon 7	chr7:140500102-140500341
7	140501167	140501286	-	*BRAF* exon 6	chr7:140501167-140501406
7	140501287	140501406	-	*BRAF* exon 6	chr7:140501167-140501406
7	140507692	140507811	-	*BRAF* exon 5	chr7:140507692-140507931
7	140507812	140507931	-	*BRAF* exon 5	chr7:140507692-140507931
7	140508624	140508743	-	*BRAF* exon 4	chr7:140508624-140508863
7	140508744	140508863	-	*BRAF* exon 4	chr7:140508624-140508863
7	140534361	140534480	-	*BRAF* exon 3	chr7:140534361-140534720
7	140534481	140534600	-	*BRAF* exon 3	chr7:140534361-140534720
7	140534601	140534720	-	*BRAF* exon 3	chr7:140534361-140534720
7	140549842	140549961	-	*BRAF* exon 2	chr7:140549842-140550081
7	140549962	140550081	-	*BRAF* exon 2	chr7:140549842-140550081
7	140624426	140624545	-	*BRAF* exon 1	chr7:140624315-140624554
12	25362668	25362787	-	*KRAS* exon 6	chr12:25362668-25362907
12	25362788	25362907	-	*KRAS* exon 6	chr12:25362668-25362907
12	25368315	25368434	-	*KRAS* exon 5	chr12:25368315-25368554
12	25368435	25368554	-	*KRAS* exon 5	chr12:25368315-25368554
12	25378508	25378627	-	*KRAS* exon 4	chr12:25378508-25378747
12	25378628	25378747	-	*KRAS* exon 4	chr12:25378508-25378747
12	25380138	25380257	-	*KRAS* exon 3	chr12:25380138-25380377
12	25380258	25380377	-	*KRAS* exon 3	chr12:25380138-25380377
12	25398144	25398263	-	*KRAS* exon 2	chr12:25398144-25398383
12	25398264	25398383	-	*KRAS* exon 2	chr12:25398144-25398383

* Biotinylated, 120-bp long ssDNA probes targeting all protein-coding exons of KRAS (exons 2-5 in NM_033360 and exon 5 in NM_004985 transcript), NRAS (exons 2-5 in NM_002524 transcript), BRAF (exons 1-18 in NM_004333 transcript), EGFR (exons 1-28 in NM_005228 transcript and exon 16 in NM_201284 and NM_201282 transcripts), and PIK3CA (exons 2-21 in NM_006218 transcript)

Supplementary Table 4. Summary of data filtering for training and validation cohorts

**Data filtering summary**	**Training Cohort**	**Validation Cohort**	**cfDNA without BM data**
Number of cfDNA samples sequenced	25	23	16
Total number of mutation calls generated by muTect	15465 (100%)	23384 (100%)	10016 (100%)
Number of mutation calls per sample, median (range)	291 (128-1614)	1273 (159-1511)	294.5 (101-1438)
Mutations removed due to sequence context bias (LOD fwd/LOD rev > |5|)	6739 (43.6%)	9553 (40.9%)	4404 (44.0%)
Mutations below LOD score threshold (modified Z-score for LOD < 20)	8548 (55.3%)	13653 (58.4%)	5493 (54.8%)
Mutations identified as germline SNPs using annotated data (Oncotator 1.5.3.0)	151 (0.98%)	149 (0.64%)	106 (1.06 %)
Likely somatic mutations detected using custom R script in R v3.2.2 (www.github.com/pughlab/lb-seq)	27 (0.17%)	30 (0.13%)	13 (0.13%)

Supplementary Methods

Yield of malignant cells from BM aspirates and quality of extracted DNA

We collected 48 BM aspirates from 40 MM patients, which were required for their clinical care or participation on the clinical trial of trametinib. From 48 BM specimens, 7 lad low yield of BM-derived malignant plasma cells prior to CD38+ cell selection (≤ 1%). As the result, the amount or quality of extracted DNA was inadequate for sequencing by the clinical laboratory. For three of these samples, we were able to sequence BM-derived tumour DNA using our 5-gene targeted sequencing method, which allowed lower input of DNA for library construction and provided increased sensitivity due to higher coverage (> 5,000X). The other four samples could not be profiled leading to screen fail (4 of 48), consistent with data published by Mulligan et al. reporting that 83-92% of BM samples passed enrichment analysis for gene expression profiling[3]. In addition, four patients were enrolled unto the CoMMpass study and three of them had BM aspirates taken to profile tumour DNA mutations. For these samples, the data on the yield of BM-derived malignant plasma cells from the BM aspirate and the quality and quantity of extracted DNA are not available. Additional 10 cfDNA samples do not have matching BM data because BM profiling was not required for their clinical care.

Comparison of BM DNA sequencing data obtained from different sources

In order to generate a list of verified tumour-specific mutations for our training and validation cohorts, we compiled genetic tumour profiling data for BM-derived DNA samples from different sources: i) BM-tumour profiling by the CAP/CLIA-certified Advanced Molecular Diagnostics Laboratory at the Princess Margaret Cancer Centre to support the trametinib clinical trial or routine clinical care of patients (31 samples, 4 of which failed); ii) BM-derived tumour DNA profiling data from patients enrolled unto CoMMpass study (4 patients), two of which had mutations data available on the MMRF's Researcher Gateway (www.themmrf.org/research-partners/the-commpass-study); and iii) data generated in our laboratory (37 samples). Samples profiled by the AMDL or CoMMpass were sequenced to at least 500X coverage using commercially available Illumina TruSeq Amplicon Cancer or TruSight Cancer Sequencing Panels, and reported tumour-derived mutations with at least 5% mutant allele frequency. In contrast, data generated in our laboratory were sequenced to at least 5,000X median coverage using the same 5-gene target capture panel used for sequencing of matching cfDNA samples, which allowed us to call tumour-specific mutation present at mutant allele frequencies below 1%. In addition, the 5-gene panel included all protein-coding exons of *KRAS*, *NRAS*, *BRAF*, *EGFR*, and *PIK3CA* and included regions not covered by the commercially available TruSeq and TruSight panels that only target the hotspot mutations in these genes, which accounted for some of the discrepancies in the sequencing data obtained from these sources. When data were available from more than one source (**Supplementary Table 1**), the preference was given to data generated using the 5-gene panel because of increased sensitivity of this method due to higher coverage. For one BM sample (from MYL-023) profiled only by the clinical laboratory, the raw sequencing data were obtained to assess mutations with allele frequencies below the clinically reported threshold of < 5%. This analysis revealed 3 additional mutations with allele frequencies ranging from 0.8% to 2.9% that were below the allele frequency threshold for reporting by the

clinical laboratory but with strong support in matched cfDNA data. One of these three mutations was a two-base substitution leading to a single amino acid protein change (*EGFR* p.A859S), and identical two-base substitution was also detected in cfDNA by LB-Seq.

Extraction of cfDNA from plasma

Within 1 hour of collection, whole blood collected in 10 mL Vacutainer Plus Plastic K_2EDTA-coated tubes (BD, Mississauga, ON) was centrifuged for 10 minutes at 1900 x g at 4°C. The plasma layer was immediately transferred to a new tube and centrifuged for 10 minutes at 16,000 g in fixed-angle rotor at 4°C. Double-spun plasma samples were stored at −80°C until DNA extraction. On the day of extraction, plasma samples were thawed at room temperature and centrifuged again for 5 minutes at 16,000 g at 4°C to remove cryoprecipitates. We extracted cfDNA from 3-13 mL of plasma (median 8.5 mL) using the QIAamp Circulating Nucleic Acid kit (Qiagen, Valencia, CA) following manufacturer’s protocol but without adding carrier RNA. Samples were eluted into 40-120 µL of elution buffer provided in the kit.

Estimation of required cfDNA input for library construction and sequencing

Tumour-derived DNA fragments make up only a small fraction (often <1%) of cfDNA in plasma, due to high concentrations of DNA fragments from non-cancerous cells, low tumour burden, or low rates of secretion of tumour fragments into the bloodstream[3]. We modeled the relationship between the potential risk of allele dropout and the amount of cfDNA used for sequencing and found that to have a 99.99% chance of capturing one tumour-derived fragment among 2,000 normal DNA fragments (allele fraction of 0.05%), we need to construct libraries from at least 83 ηg of cfDNA (**Fig. 2** of the paper). Therefore, to minimize the risk of allele dropout, we chose to prepare cfDNA-sequencing (cfDNA-seq) libraries using 83 ηg of cfDNA or the maximum amount available, if less than 83 ηg. By extrapolating cfDNA yields from our MM cohort, we estimated that this quantity of cfDNA could have been attained from all patients through collection of 18 mL of blood plasma (**Supplementary Fig. 1**). Of note, cfDNA yields from plasma of MM patients were higher compared to patients with other advanced solid tumours (listed in **Supplementary Table 2**), which were also processed in our laboratory using the same method (**Fig. 1a**), suggesting that the volume of plasma required for obtaining 83 ηg of cfDNA may also be higher in other cancers.

Preparation of cfDNA-sequencing (cfDNA-seq) and BM DNA sequencing libraries

We prepared cfDNA-seq libraries using KAPA Hyper Prep Kit for Illumina TruSeq library construction (Kapa Biosystems, Wilmington, MA) following manufacturer’s protocol. CfDNA fragments circulate as short oligomers with fragment size corresponding to the length of double-stranded DNA loops around one, two, three, or more nucleosomes plus intervening linker sequences (approximately 166 bp, 332 bp, 498 bp, etc. respectively)[4,5]. To preserve the natural size distribution of cfDNA fragments, we prepared cfDNA-seq libraries without additional fragmentation. BM-derived DNA samples were sheared to target 300 bp fragment size (approximate range 100-700 bp).

DNA sequencing libraries from all cfDNA and BM samples processed in our laboratory were then produced using similar laboratory workflow, as described in **Supplementary Fig. 2**. We

used two types of single-indexing adapters, Illumina TruSeq LT adapters, 6 and 8 nucleotide index (Illumina, San Diego, CA) or Illumina-compatible 8 nucleotide index NEXTflex-96 DNA Barcodes (Bioo Scientific, Austin, TX). Depending on the total yield of cfDNA from each sample, we prepared libraries using 83 ηg of input DNA (51 of 64 samples) or as much cfDNA as was available (10-80 ηg, 13 of 64 samples). For BM-derived tumour DNA samples, 83 ηg of sheared genomic DNA was used for library construction.

To test the effect of using lower cfDNA input for library construction, we compared the efficiency of cfDNA-seq library construction using Illumina LT (**Supplementary Fig. 3a**) or NEXTflex (**Supplementary Fig. 3b**) sample barcodes and 83 ηg or lower amount of input cfDNA. Pre-capture libraries were amplified using 4-8 cycles of PCR using Illumina P5 and P7 primers and a high-fidelity low-bias PCR amplification kit, the KAPA HiFi HotStart ReadyMix (Kapa Biosystems, Wilmington, MA). Post-ligation and post-amplification magnetic bead clean-up was performed by Agencourt AMPure XP PCR Purification system (Beckman Coulter, Brea CA) using 0.8X or 1X SPRI beads, respectively, according to KAPA Hyper Prep kit protocol. DNA fragments bound to magnetic beads were extracted from solution using a magnetic tube rack (DynaMag-2, Life Technologies, Burlington, Ontario, Canada) and eluted into 1X TE buffer consisting of 10 mM Tris and 1 mM EDTA, pH 7.5 (post-ligation) or nuclease-free water (post-amplification).

Strikingly, we observed significantly lower sample recovery after adapter ligation, PCR-amplification, and bead clean-up for low input libraries compared to high input libraries (12% vs. 25%, $P < 0.001$) when using Illumina LT adapters (**Supplementary Fig. 3a**). This may be in part due to increased number of amplification cycles used for 10 ηg input versus 83 ηg input samples (8 cycles versus 4 cycles). For six of seven of the low-DNA input samples, we prepared new libraries using the remaining 64-83 ηg of input DNA, which were used for downstream target capture and sequencing. MYL-013 cfDNA sample was sequenced using library generated from 10 ηg of cfDNA due to lack of material. For samples barcoded using NEXTflex adapters (**Supplementary Fig. 3b**), we used 83 ηg of cfDNA (32 samples) or maximum available (34-79 ηg, 7 samples). Similarly to Illumina LT adapters, higher DNA input was associated with better sample recovery after adapter ligation, PCR-amplification, and bead clean-up (32% vs. 43%, $P = 0.031$). In addition, for libraries made with 83 ηg input we observed higher sample recovery when using NEXTflex adapters compared to Illumina adapters (25% vs. 43%, $P < 0.001$). However, we cannot be confident that these changes are due to the differences in adapter type alone, as we did not conduct a direct comparison of the two adapter types in a well-controlled experiment.

Hybrid capture and enrichment of target genomic regions

To enrich cfDNA-seq libraries for genomic regions of interest, we designed a custom IDT xGen Lockdown target capture panel for ultra-deep targeted sequencing of cfDNA that targeted 11 kb of the genome encoding all protein-coding exons of *KRAS*, *NRAS*, *BRAF*, *EGFR*, and *PIK3CA*. This target capture panel included 146 synthetic, 120 nt-long ssDNA biotinylated capture probes targeting all exons of *KRAS*, *NRAS*, *BRAF*, *EGFR*, and *PIK3CA* with 1X tiling density (**Supplementary Table 3**, xGen Lockdown Custom Probes Mini Pool, Integrated DNA Technologies (IDT), Coralville, IA). To streamline laboratory workflows and enable multiplex sequencing, we pooled 8-10 cfDNA-seq libraries with unique sample

barcodes in equal concentrations (500-1000 ηg of total pooled DNA) prior to hybrid capture. To block adapter sequences from binding each other during hybridization and target capture, we added universal blocking oligos, which varied depending on the barcode sequences used for library construction. For libraries prepared using NEXTflex-96 DNA Barcodes, pooled DNA was combined with 5 μg of human Cot-1 DNA (Invitrogen), 1 μL of TS-P5 xGen Universal Blocking Oligos, and 1 μL of 8 nucleotide-index TS-p7 xGen Universal Blocking Oligos (IDT, Coralville, IA). For cfDNA-seq libraries prepared using Illumina TruSeq LT adapters, sample preparation was similar with the exception of using 1 μL of a mixture of 6 and 8 nucleotide index TS-p7 xGen® Universal Blocking Oligos in appropriate proportion reflecting the relative numbers of samples ligated to 6 and 8 nucleotide index TruSeq LT adapters within the pool.

Pooled libraries, blocking oligos, and human Cot-1 DNA were dried down using a vacuum centrifuge (Eppendorf SpeedVac concentrator) and resuspended in 7.5 μL NimbleGen Hybridization Buffer, 3 μL NimbleGen Hybridization Component A (SeqCap EZ Hybridization and Wash Kit, Roche NimbleGen, Madison, WI), and 2.5 μL nuclease-free water. The hybridization mix was then incubated in a thermal cycler at 95°C for 10 minutes. Immediately after 95°C incubation, 3 ρmol of xGen Lockdown Probes pool (resuspended in 2 μL of TE buffer) was added to the hybridization solution (17 μL final volume), followed by 16-20 hour hybridization at 65ºC in a thermal cycler (Applied Biosystems Veriti 96-Well Thermal Cycler, model 9902, Life Technologies, Burlington, Ontario, Canada). After hybridization, target DNA fragments hybridized to complementary biotinylated probes were captured from solution using 100 μL of biotin-binding Streptavidin Dynabeads M-27 (Life Technologies, Burlington, Ontario, Canada). After 45 min incubation in the thermal cycler, streptavidin beads bound to target DNA were extracted from solution using DynaMag-2 magnetic tube rack. The SeqCap EZ Wash Kit was used to prepare Streptavidin beads for target capture and remove off-target DNA fragments from within the pooled sample according to manufacturer protocol. Due to the small size of the target region (0.0006% of human genome), we had to amplify cfDNA-seq libraries post-capture using 13-15 cycles of PCR to generate sufficient amount of DNA for quality control assessment and sequencing. The post-capture PCR amplification was performed using the KAPA HiFi HotStart ReadyMix and Illumina P5 and P7 primers (50 μL reaction volume), with streptavidin beads kept in solution during PCR. Amplified targeted cfDNA-seq libraries were extracted from solution using 1.5X SPRI beads (Agencourt AMPure XP PCR Purification system) and eluted in 22 μL of 1X TE buffer.

DNA quantification and fragment size distribution analysis

To ensure consistent tracking of library quantity and quality throughout the process, we profiled DNA concentration and size distribution for initial cfDNA extractions, each cfDNA-seq library prior to hybridization and target capture, and the final pooled capture library. DNA concentrations were measured using the Qubit dsDNA HS Assay Kit (Life Technologies, Burlington, Ontario, Canada). Fragment size distributions were assessed by Agilent 2200 TapeStation system using the Agilent Genomic DNA Screen Tape for original cfDNA samples or the High Sensitivity D1000 Screen Tape (Agilent Technologies, Santa Clara, CA) for DNA-seq libraries and pooled captured DNA-seq libraries. DNA fragment size distribution in the cfDNA-seq library (**Supplementary Fig. 4a**) was similar to the corresponding extracted cfDNA sample (**Supplementary Fig. 4b**), with expected shift in molecular weight due to the

addition of sequencing adapters. Similarly, the insert size inferred from mapping of DNA sequencing reads, which was assessed using (Picard CollectInsertSizeMetrics tool)[2], reflected the original distribution of cfDNA fragment sizes, demonstrating preserved complexity of cfDNA-seq libraries (**Supplementary Fig. 4c**).

Prior to sequencing, library size and fragment size distribution of the pooled captured DNA samples were evaluated using Agilent High Sensitivity DNA Kit and reagents using Agilent 2100 Bioanalyzer (Agilent Technologies, Santa Clara, CA) according to manufacturer protocol. Captured DNA library concentrations were then verified by qPCR at 1:4000 and 1:8000 dilutions using KAPA Library Quantification Kit for NGS (Kapa Biosystems, Wilmington, MA) on the CFX96 Touch Real-Time PCR Detection System (Bio-Rad, Hercules, CA) according to manufacturer recommendations.

Next-generation sequencing of captured cfDNA-seq libraries

Captured target DNA fragments from pooled cfDNA-seq libraries were sequenced on Illumina HiSeq 2000 instruments using TruSeq PE Cluster Generation Kit v3 for clustering, with 1% phiX spike-in as a quality control, and TruSeq SBS Kit v3 for 100-bp paired-end sequencing according to manufacturers instruction but using a custom sequencing run (read 1, index 7, index 5, and read 2 for 101, 9, 0, and 101 cycles, respectively). Five cfDNA libraries were sequenced on Illumina HiSeq 2500 using TruSeq PE Cluster Generation Kit v4 and TruSeq SBS Kit v4 and similar conditions. By using a small target capture panel (146 baits covering genomic region of 18 kb), we were able to achieve mean bait coverage of >20,000X for all cfDNA samples by pooling 20-25 samples per flow cell lane on a HiSeq2000 instrument. The mean bait coverage was estimated using the GATK Depth of Coverage tool, counting the number of fragments rather than the number of reads, as the later overestimates the coverage by 5-25% due to counting overlapping paired-end reads twice (for read fragments shorter than 200 bp). Samples with initial sequencing mean bait coverage < 20,000X but adequate enrichment of target regions (i.e., ≥ 40% of reads on or within 200 bp of the target probe), which included two cfDNA samples from MYL-033 and 036, were sequenced again as a batch (all samples pooled prior to target capture had to be resequenced). Raw alignments were merged, if necessary, to achieve > 20,000X mean bait coverage (alignments merged only for MYL-033 and 036 but not the other samples in the batch). Libraries with inadequate enrichment of target regions (i.e., < 40% of reads on or within 200 bp of the target probe) were pooled and captured again and resequenced to achieve mean bait coverage > 20,000X (included MYL-033(2), 039, and 040). For these samples, only the new data with adequate target enrichment were used for sequence analysis. In the final dataset, desired mean bait coverage > 20,000X was achieved in all cfDNA samples (**Supplementary Fig. 5a**). Target regions were enriched 64,800-112,000 fold over background genomic sequence, with median 70% of reads on or within 200 bp of a hybrid-capture probe (range 49-80%) (**Supplementary Fig. 5b**).

Estimation of duplication rate and loss of unique cfDNA molecules during processing

For a typical cfDNA sample, 83 ηg of cfDNA was used to prepare adapter-ligated sequencing library. Since cfDNA is naturally fragmented with a major fragment size corresponding to a DNA loop around one nucleosome (~166 bp, 80-85%) and a second peak (~15-20%)

corresponding to two nucleosomes (332 bp), we estimated the average fragment size of cfDNA to be approximately 180 bp. Using 650 Daltons as an average weight of one DNA base pair, we estimated that 83 ηg of cfDNA contains approximately 4.3×10^{11} unique DNA fragments (7.1×10^{-13} mol). For libraries prepared using 83 ηg of input cfDNA (n=33), the library recovery after ligation to NEXTflex-96 adapters and four amplification cycles was median 43% of the theoretical maximum yield (21-70% range, **Supplementary Fig. 3b**). Some of this reduction in yield is due to loss of unique DNA templates during adapter ligation and initial bead clean-up, and some is likely caused by inefficiencies in post-ligation PCR and final library clean-up, primarily causing loss of redundant library fragments without major effect on library complexity. If we presume that the entire reduction in library yield is due to loss of unique DNA templates (the most conservative estimate of library complexity), the estimated number of unique cfDNA molecules converted into a barcoded DNA-seq library is 1.8×10^{11} ($0.88–3.0 \times 10^{11}$). Since the target region (146 x 120 bp probes = 17.5 kbp) makes up only 0.0006% of the genome, we estimated that a similar proportion of cfDNA molecules present in each sample will align to the region of interest and will be captured for sequencing (1.1×10^{6} unique cfDNA fragments). Since ligated fragments within the library are amplified 4 PCR cycles (theoretically resulting in 16 copies of each molecule within the pool), the downstream steps are less likely to result in complete allele drop out, despite known inefficiencies in hybridization of target probes to DNA fragments, capture of hybridized DNA probes, post-capture PCR, or magnetic bead clean-up of amplified DNA product. Not all cfDNA fragments containing target DNA sequence will bind to their complementary DNA-binding probes, some of the hybridized DNA will not be captured by streptavidin beads or will be lost during clean-up, and each cycle of PCR amplification after target capture will introduce additional loss of DNA material as well as potential amplification bias.

Given the small size of our target capture panel (17.5 kb), we only quantified the final amplified and purified DNA product prior to sequencing, but did not quantify DNA yield immediately after target capture but prior to amplification, which would result in substantial loss of captured DNA material. Without this intermediate quantification or the use of molecular barcoding strategies[6–8], it is difficult to estimate the proportion of DNA molecules lost during each step involved in target capture protocol.

In light of these limitations, we have taken several measures to improve our target capture efficiency such as i) using individually synthesized high quality DNA binding probes (designed, synthesized, and individually tested by the Integrated DNA Technologies); ii) using an abundance of target capture probes (0.002 ρmol of each probe) per target capture reaction; and iii) increasing the hybridization time from 4 hours to 16-20 hours (overnight) at 65°C in order to improve probe hybridization to target DNA. Our protocol also allows direct PCR amplification of captured DNA without intermediate DNA clean-up, with streptavidin beads left in solution along with captured DNA templates during 13-15 cycles of PCR amplification, which reduces the loss of captured DNA templates. For all PCR steps in our protocol, we used KAPA HiFi polymerase high fidelity enzyme, which has been shown to improve amplification efficiency and reduce amplification bias for extremely GC-rich or AT-rich fragments[9]. Together with the redundancy of each unique DNA template present in the library pool, we believe that the inefficiencies during hybridization, target capture, and post-capture PCR are unlikely to lead to significant reduction in library complexity.

A study by Newman et al. used molecular barcode-ligated cfDNA libraries and the mark and recapture method to estimate that approximately 50-60% of total human genome equivalents which enter into the library preparation are present after target capture and post-capture PCR (~40-50% loss of unique cfDNA templates)[6]. Although the methodology used in our study was different (e.g., using sample barcodes and a different target capture panel), we anticipate our rates of recovery of unique cfDNA fragments during sample processing to be similar to these published data.

Assuming 180 bp to be the average size of cfDNA fragments, approximately 98 fragments will be required to achieve 1X coverage of the 17.5 kbp target region. Using our conservative estimates that 1.1×10^6 unique cfDNA fragments will align to the target region and will be captured for sequencing, we estimate that the median depth of coverage that can be achieved through sequencing of 1.1×10^6 unique cfDNA fragments is approximately 11,000X. Hence, mutations detected in a region with 20,000X coverage should have ~55% unique reads, and this fraction will decrease in regions with higher coverage (~11% unique reads for regions with 100,000X coverage). Higher degree of duplication is likely to be present in samples with lower initial cfDNA input into library constructions or in libraries prepared using Illumina LT adapters (the ligation efficiency was lower compared to NEXTflex adapters, **Supplementary Fig. 3**). If the duplication rate is similar between normal and tumour-derived cfDNA fragments during sample processing, the AFs observed for tumour-derived mutations remain largely independent of the duplication rate and representative of the allele fractions present in plasma.

LB-Seq bioinformatics pipeline

Detection of likely somatic mutations in cfDNA is challenged by the need to distinguish germline variants from bona fide somatic mutations and the presence of low-frequency (<0.2%) base substitution artifacts introduced by polymerase error during library construction and subsequent Illumina sequencing and errors during sequencer base calling[10]. To address these problems, we developed an algorithm for identifying likely somatic mutations in cfDNA sequencing data without matching normal (**Supplementary Figs 6** and **7**). Initially, raw sequencing data were de-multiplexed using the sample-specific barcode adapters to generate sample-specific FASTQ files. Reads were aligned to the human genome reference (Illumina iGenomes hg19) using bwa[11] version 0.7.12 and post-processed following Genome Analysis Toolkit (GATK) Best Practices[1] but without marking duplicates (**Supplementary Fig. 6**). Following initial processing of cfDNA sequencing data, all candidate point mutations were identified by muTect version 1.1.4[12] and annotated using Oncotator version 1.5.3.0[13].

Due to the natural fragmentation of cell-free DNA, with predominant fragment size corresponding to the DNA loop around one nucleosome, the likelihood of mistakenly reducing the true library complexity by removing duplicates is greater than for sheared genomic DNA. In our training cohort that included 25 cfDNA samples with matching BM-derived tumour DNA sequencing data with a total of 26 mutations detected in the BM samples, when we marked duplicates in cfDNA data, only 10 of 26 tumour-derived mutations (38% concordance with BM data) were detectable by LB-Seq without any false positives (100% specificity). In contrast, 25 of 26 mutations (96% concordance with BM data) were detectable in the same cfDNA dataset when duplicates were not marked, and specificity was again 100% (one apparent

false positive call is believed to be a real mutation only detectable in cfDNA but not BM of MYL-001 patient). All mutations with allele frequencies < 5% were missed when using GATK marked duplicates processing and typically 80-95% of the sequencing data were discarded as duplicate reads. This demonstrated that marking duplicates using the standard tools is reducing our ability to detect real mutations in cfDNA data and diminishes the sensitivity of this method. Thus, we followed the procedures described by Newman, Bratman et al. in *Nature Medicine*[14], which also determined that sensitivity for rare variant detection within cell-free DNA was improved when duplicates were retained. Thus, we concluded that it would be inappropriate to perform deduplication using GATK mark duplicates tool or similar tools that rely only on identical genomic position and fragment size. To account for sequencing artifacts, we have implemented a downstream analysis pipeline that allows us to remove false positive mutation calls based on tumour LOD scores. Furthermore, muTect eliminates mutation calls that come from the same read position (i.e., potential duplicate fragments). These mutations are rejected by muTect due to "clustered_read_position" determined based on having a very low median absolute deviation in read position of the base substitution within each fragment (--pir_mad_threshold of 3 or lower, the default setting). We used the default parameters to identify such artifacts, so many mutation calls based solely on data from the same or clustered read position were rejected. We feel that our results (>98% specificity and 96% concordance with mutations detected in the BM samples) demonstrate that this analysis pipeline can adequately distinguish between true mutations and sequencing artifacts, despite keeping some duplicate reads in the data.

Detection of low frequency mutations in cfDNA deep sequencing data

When we initiated this study, bioinformatics tools for discovery of ultra-rare mutations (with allele fractions < 1%) in cfDNA deep sequencing data (>20,000X) were lacking. MuTect is a widely used bioinformatics tool for calling single nucleotide variants (SNVs) and is currently recommended by the Genome Analysis Toolkit Best Practices document for mutation detection in exome, whole genome, and targeted panel sequencing studies (https://software.broadinstitute.org/gatk/best-practices/). In our study, we explored the utility of using muTect for analysis of low frequency (<1%) mutations in ultra-deep cfDNA sequencing data (>20,000X) and without matching normal using adjusted configuration. The configuration changes we implemented included reducing "fraction_contamination" threshold to 0, increasing "gap_events_threshold" to 1000 due to increased target coverage in cfDNA data, as well as using "force_alleles" argument to prevent rejection of mutations due to triallelic sites (**Supplementary Figs 6b** and **7**).

Some of the changes made in muTect parameters generated new analysis issues that needed to be resolved in downstream filtering. One such issue was related to using "force_alleles" argument during muTect analysis. The default settings allow only one variant with the highest tumour LOD score to be called at any particular locus. Forcing alleles at triallelic sites allows detection of up to three independent single-base substitutions (e.g., A>C, A>G, and A>T) at the same genomic locus. This is important for cfDNA data analysis since overlapping mutations at the same locus may originate from distinct tumour DNA fragments derived from different clonal populations. However, this adjustment led to some false mutation calls with apparently high AFs at loci where homozygous, non-reference sequence SNPs were present. Because 90-99.9% of the reads at these loci typically corresponds to the

non-reference allele (i.e., homozygous SNPs), the reference allele count at these lcan be very low. MuTect analysis calculates tumor_f values independently for each substitution (tumor_f = t_alt_count/(t_ref_count + t_alt_count)), with denominator being the sum of alternative and reference allele counts and not the total read count at that locus. At homozygous SNP loci, this leads to overestimation of the AFs for the remaining two possible single-base substitutions and results in uncharacteristically high tumor_f and tumour LOD scores (dependent on tumor_f) for these variant calls. To avoid calling these erroneous calls as real mutations, we have removed any mutations for which the sum of reference and alternative allele counts equals to < 10% of total reads. This threshold was selected based on the assumption that at homozygous SNP loci, ≥ 90% of the reads will correspond to the non-reference SNP alternative allele, leaving <10% of reads for the reference allele and the other two possible alternative variants. These mutation calls were removed prior to converting the tumour LOD scores into the modified Z-scores to avoid skewing the LOD-score distribution using these low confidence error calls with apparently high LOD scores.

Custom data filtering algorithm to distinguish real biologic variants from sequencer artefacts or polymerase errors

Candidate mutations that passed muTect analysis (“judgement” = KEEP) were further filtered using our custom data-filtering algorithm developed in R version 3.2.2 (available at www.github.com/pughlab/lb-seq) using muTect and Oncotator output data. This algorithm aims to differentiate real genetic variants present at very low allele frequencies from sequencing artifacts or polymerase errors with similar apparent allele frequencies. For this purpose, we used muTect-derived tumour LOD scores - a statistical measurement of the likelihood that the mutation is a true biologic variant and not a sequencer artifact or an error introduced by polymerase[12]. To account for variability of the absolute tumour LOD score values between samples and sequencing batches (**Fig. 4** in the main text), tumour LOD scores were transformed into modified Z-scores, i.e. the distance from the median divided by the median absolute deviation (MAD)[15], using the distribution of tumour LOD scores within each sample. The use of median and MAD for transforming these data was necessary due to highly skewed (not normal) distribution of LOD scores in each sample, and a similar approach has been described previously for transformation of high throughput screening data generated for large-scale RNA interference libraries[16]. We then removed context-specific sequencing artifacts, defined as mutations with > 5-fold difference in forward and reverse LOD scores (i.e., only kept mutations with |t_lod_fstar_forward / t_lod_fstar_reverse| ≤ 5), from the dataset. To further differentiate real mutation calls from the hundreds of low allele frequency sequencing artifacts remaining in the data, we compared modified Z-scores (transformed tumour LOD score values) for true positive (TP) and false positive (FP) mutations using 25 cfDNA samples with matched BM-derived DNA sequencing data available (training cohort). A total of 27 single-base substitutions were identified in this set of 25 BM samples and these mutations were then used to classify the candidate mutation calls in matching cfDNA samples as real (TP, if identical) or false (FP, if not found in the matching BM). Mutations that were detected in the BM but were not detected in cfDNA were classified as false negative (FN). These data were then used to generate a Receiver Operator Curve (ROC) correlating the modified Z-score used as a threshold for calling real mutations and the corresponding False Positive and False Negative rates (**Supplementary Fig. 9**). This ROC

curve was then used to select a modified Z-score threshold for calling likely somatic mutations in cfDNA data (**Supplementary Fig. 9b**). When using modified Z-score = 20 as a threshold, 26 of 27 true positive calls are detected in cfDNA (96.3% concordance with BM sequencing data) and the only apparent "false positive" mutation call (*PIK3CA* p.Y207* mutation in MYL-001) we believe is a real mutation identified only in cfDNA (at AF = 0.28%) but not represented in the corresponding single-site BM aspirate. By increasing modified Z-score threshold to 40-43, this apparent "false positive" call can be eliminated; however, only 22 of 27 true positives will be identified (81.5% concordance with BM sequencing data). Hence, the modified Z-score value of 20 (i.e., tumour LOD score that is 20 MADs higher than the median in each sample) was selected as the optimal threshold value to maximize sensitivity and specificity of the LB-Seq method.

Approach for identifying non-pathogenic germline polymorphisms without sequencing the matching normal DNA

We developed an algorithm that would allow us to differentiate between germline SNPs and tumour-derived somatic variants without sequencing the matching normal DNA. Given the availability of many large databases of population sequencing data (e.g., dbSNP, 1000Genomes, ExAC, and ClinVar), we chose not to sequence matching normal samples from each patient to reduce the cost of sequencing. Among the "real" genetic variants which passes the first stage of our filtering algorithm, non-pathogenic germline SNPs were selected using population data from large databases such as dbSNP (build 142) and the 1000 Genomes Project (1000gp3 20130502 database), using annotation data generated by Oncotator version 1.5.3.0 to streamline the analysis[13]. As a safeguard against incorrectly labelling tumour-derived somatic variants as germline SNPs, this script classifies a variant as germline polymorphism only if dbSNP classification of this variant as a validated SNP is supported by at least 1 in 1000 frequency in 1000 Genomes Project (i.e., the variant has population frequency > 0.1% in the 1000gp3 20130502 database). In addition, to account for germline SNPs that are recurrent in specific ethnic populations (e.g., American, African, European, South Asian, or East Asian), we also labeled variants as germline SNPs if they had allele frequency >0.1% in any of these ethnic populations. This approach is based on an assumption that if a particular variant occurs in healthy adults of particular ethnicity, it is more likely to be a germline SNP than a somatic tumour-derived mutation. By applying the described approach for identifying germline SNPs to data annotated using Oncotator version 1.5.3.0, we were able to remove germline SNPs from our list of candidate mutations. By exclusion, all other "real" mutation calls that passed LOD score filtering algorithm were labelled as likely somatic variants. This approach allowed us to differentiate between germline SNPs and likely somatic tumour-derived variants without sequencing the matching normal DNA. However, for larger panels or patients with evidence of high AF mutation calls (>40%) we recommend sequencing matching germline DNA, which can be obtained from leukocytes readily available from the same blood samples used for cell-free DNA extraction.

Comparison of cfDNA and BM-derived tumour DNA mutation calls

Mutations detected in cfDNA sequencing data were compared to the mutations in the corresponding BM-derived DNA sequencing data from matched BM samples (from BM

aspirates collected within 1 week of the blood draw) based on exact match in the genomic substitution detected in both samples. Each matching genomic substitution between cfDNA and BM-derived DNA sequencing data was counted as a true positive mutation call. Mutations detected only in the BM-derived tumour DNA specimen but not in the corresponding cfDNA sample were reported as a false negative. Mutations detected only in the cfDNA but not in the matching BM-derived tumour DNA sample were reported as false positive, unless the BM data did not cover this particular mutation (e.g., two *PIK3CA* mutations in MYL-001(2) were not tested by commercially available panels used to sequence the BM sample in the clinical lab). If the same gene was called devoid of mutations by cfDNA and BM sequencing, it was counted as a single true negative. All protein changes reported were caused by a single-base missense mutation, with the exception of *EGFR* p.A859S mutation in MYL-023 and *KRAS* p.A59L mutation in MYL-029, both of which were associated with a two-base substitution (chr7: 55259516-55259517 GG>CT and chr12: 25380282-25380283 GC>AA genomic change, respectively). Two patients had multiple mutations within the same gene, i.e., three *KRAS* mutations in MYL-020 and two *NRAS* mutations in MYL-028 (**Fig. 5c**), which were each counted as a true positive result. The sensitivity (SN), defined as concordance with BM sequencing data, specificity (SP), positive predictive value (PPV), and negative predictive value (NPV) calculated for each cohort (training and validation) and for all data combined are shown in **Supplementary Fig. 10**.

Validation of discordant mutation calls by ddPCR

For three discordant calls present in the training and validation datasets: *KRAS* p.G12D mutation not detected in MYL-020-cfDNA, *KRAS* p.G12V mutation not detected in MYL-054-cfDNA, and *PIK3CA* p.E545K mutation not detected in MYL-049-BM (**Supplementary Fig.11a**, **b**, and **c,** respectively), we confirmed the results from LB-Seq analysis by droplet digital PCR (ddPCR). For ddPCR analysis, we used PrimePCR™ ddPCR™ Mutation Assays obtained from Bio-Rad Canada (Mississauga, Ontario, Canada) that were designed and validated for rare-variant detection analysis. For each assay, we used 25-50 ŋg of input DNA, either extracted DNA material (MYL-049-BM, MYL-049(2)-cfDNA) or the corresponding adapter-ligated library DNA (MYL-020-cfDNA-lib, MYL-054-cfDNA-lib, MYL-049-cfDNA-lib, and MYL-049(2)-cfDNA-lib). HEX-labelled probe for WT allele and FAM-labeled probe for mutant allele were combined with sample DNA and other ddPCR reagents prior to generating droplets using QX200™ Droplet Generator (Bio-Rad), according to manufacturer protocol. After PCR amplification of input DNA on C1000Touch™ Thermal Cycler (Bio-Rad), the fluorescence signal for each probe was simultaneously measured in each droplet using QX200™ Droplet Reader (Bio-Rad) and QuantaSoft™ version 1.7.4 to assess mutant allele fractions. A different positive control was used for each assay in **Supplementary Fig. 11**: MYL-015-cfDNA for *KRAS* p.G12D (**a**), MYL-058-cfDNA for *KRAS* p.G12V (**b**), and MYL-049-cfDNA or MYL-049(2)-cfDNA for *PIK3CA* p.E545K (**c**). MYL-021-cfDNA sample was used as a negative control in all three assays. For positive control samples, the mutant AFs detected by ddPCR were in agreement with AFs obtained from LB-Seq analysis. Similarly, the negative control, MYL-021-cfDNA, had undetectable signal for each mutation, confirming the absence of these mutations reported by LB-Seq. We also demonstrated that using cfDNA-sequencing library as input for ddPCR analysis produced the same results as using original cfDNA sample in all cases tested: MYL-021-cfDNA vs. MYL-021-cfDNA-lib; MYL-015-cfDNA vs. MYL-015-cfDNA-lib; MYL-058-cfDNA vs. MYL-058-cfDNA-lib; and MYL-

049(2)-cfDNA vs. MYL-049(2)-cfDNA-lib. Hence, when original cfDNA sample was used up (MYL-020-cfDNA, MYL-054-cfDNA, and MYL-049-cfDNA), we used the corresponding cfDNA-seq library as a surrogate source of DNA for ddPCR analysis. The discordant negative result detected by LB-Seq for *KRAS* p.G12D mutation in MYL-020-cfDNA, *KRAS* p.G12V mutation in MYL-054-cfDNA, or *PIK3CA* p.E545K mutation in MYL-049-BM (**Supplementary Fig.11a**, **b**, or **c**, respectively) was confirmed by ddPCR analysis.

Correlation between mutant allele fractions in cfDNA and BM-derived tumour DNA

When looking across all cfDNA sequencing data with matching BM analysis, mutant allele fractions observed in cfDNA did not correlate with the allele fractions detected in the matching BM samples ($R^2 = 0.321$, **Supplementary Fig. 14**). However, when we grouped the data for patients with multiple likely somatic mutations, which included twelve samples from nine patients (**Fig. 5** of the main text and **Supplementary Figs 15 and 16**), we observed a high degree of correlation between the BM and cfDNA allele fractions for each set of mutations detected in each sample pair (**Fig. 5a**), with R^2 values ranging between 0.913 and 0.997 for four sample pairs with at lease three mutations detected in cfDNA analysis. Furthermore, we identified two patients with mutations in three different genes, *KRAS*, *BRAF*, and *EGFR*, with similar subclonal hierarchy determined from cfDNA and BM tumour DNA sequencing (**Fig. 5b**). In addition, two patients had multiple mutations within the same gene, i.e., three *KRAS* mutations in MYL-020 and two *NRAS* mutations in MYL-028 (**Fig. 5c**). MYL-028 had two distinct *NRAS* missense mutations affecting the same protein amino acid (glutamine 61), which were caused by two separate single-base substitutions of adjacent bases. By looking at the aligned sequencing data for the matched cfDNA and BM-derived DNA samples, we observed no overlap between these two substitutions, leading us to rule out a two-base substitution and report two distinct missense mutations (p.Q61R and p.Q61K) that likely represent two distinct tumour subclones (**Supplementary Fig. 17**). In three patients with serial blood draws (MYL-001, 012, and 049), we observed similar consistency of mutational hierarchy between cfDNA and BM-derived DNA samples (**Supplementary Fig. 15**). In two patients, cfDNA analysis uncovered two different *PIK3CA* variants not found in matched BM but consistent across serial blood draws (p.Y207* in MYL-001 and p.E545K in MYL-049, **Supplementary Fig. 15a,c**) and not detected in any other cfDNA samples examined in this study. Hence, we concluded that these are real tumour-derived mutations that were detected by LB-Seq but missed by BM sequence analysis. MYL-012 patient had identical *KRAS* (p.A146V) and *BRAF* (p.D594G) mutations detected in cfDNA and BM-derived DNA. Surprisingly, the AF for the *BRAF* mutation was higher than *KRAS* in the BM in both serial samples, whereas similar AFs for both mutations were detected in serial cfDNA samples (**Supplementary Fig. 15b**). Two other samples with multiple mutations, MYL-007 and 044, both had *KRAS* and *EGFR* mutations, with similar clonal hierarchies detected by cfDNA and BM sequencing (**Supplementary Fig. 16a,b**), while for MYL-063 patient, *KRAS* and *EGFR* mutations were identified in cfDNA but only *KRAS* mutation was detected in the matching BM tumour DNA (**Supplementary Fig. 16c**). This mutation has been previously reported in lung cancer[17] and may represent a true mutation missed by BM sequencing. Finally, MYL-070 patient had *KRAS* and *BRAF* mutations, with similar clonal hierarchies detected by cfDNA and BM sequencing (**Supplementary Fig. 16d**).

Supplementary Note 1. Correlation of clinical course with longitudinal monitoring of likely somatic genomic alterations in plasma cfDNA

Patient MYL-001: A 59-year old female with multiply relapsed MM, who was progressing on a clinical trial of bortezomib combined with dexamethasone and the insulin growth factor 1 receptor (IGF-1R) inhibitor, linsitinib, at the time of blood and BM sampling. Free kappa light chain measured 2002 mg/L at the time of initial cfDNA sampling. She was then started on pomalidomide and dexamethasone. After 28 days of pomalidomide and dexamethasone her free kappa light chains had increased to 11043 mg L^{-1}, while free lambda light chain levels remained constant (0.7 mg/L), suggesting progression. Clinical profiling of the two serial BM samples identified only *NRAS* p.Q61K mutation with allele fraction of 47% in both BM samples (**Supplementary Table 1**). *NRAS* p.Q61K mutation was also detected in cfDNA at 32% allele fraction. We also uncovered two additional mutations in *PIK3CA* p.I841V and p.Y207*, respectively, but only *PIK3CA* p.I841V could be detected in the BM using our five gene panel (**Fig. 3**, **Supplementary Table 1**). At progression, *NRAS* p.Q61K and *PIK3CA* p.I841V mutations remained relatively stable in cfDNA (AFs varied from 32% to 26% and from 23% to 22%, respectively), while the *PIK3CA* p.Y207* AF increased from 0.28% to 1.4% with progression.

Patient MYL-003: A 54-year old female with multiply relapsed MM had *NRAS (*p.Q61K*)* mutation in tumour tissue with an AF of 44% at screening for the PHL-9460 clinical trial of trametinib. Blood obtained for cfDNA analysis after two months of single agent trametinib treatment demonstrated the presence of an *NRAS* (p.Q61K) mutation with an AF of 1.3% suggesting a clonal regression. Coincidently the M spike had decreased from 52 to 31.3 g L^{-1} demonstrating a response to treatment. After 9 months on treatment protocol, the patient progressed with an increase in the M spike from a nadir to 15.2 to 32.5 g L^{-1}. After discontinuation of trametinib, the M spike, cfDNA and tumour DNA AFs all increased rapidly to 48.5 g L^{-1}, 20% and 61% respectively (**Fig. 4c**).

Patient MYL-008: A male with multiply relapsed MM on treatment with pomalidomide, dexamethasone and bortezomib had no evidence of mutations in *NRAS, KRAS, BRAF, PIK3CA* or *EGFR* by cfDNA sequence analysis. Two months later, the free lambda light chains had significantly increased. The cfDNA analysis showed no evidence of clonal evolution with respect to *NRAS, KRAS, BRAF, PIK3A* or *EGFR* associated with this patient's progressive disease; however, we observed an increase in yield of cfDNA from 11 to 17.5 ηg mL^{-1}.

Patient MYL-012: A 72-year old female with multiply relapsed MM had *KRAS* (p.A146V) and *BRAF* (p.D594G) mutations in tumour tissue and cfDNA with AFs of 8.0% and 7.6% (*KRAS*) and 14% and 8.2% *(BRAF),* respectively, prior to starting treatment with pomalidomide, carfilzomib and dexamethasone (PCD). She experience disease progression after 10 months on this treatment. Shortly thereafter she was enrolled onto the PHL-9460 clinical trial of trametinib. Screening tumour and cfDNA analysis for stratification to biomarker positive or negative groups of the PHL-9460 study demonstrated no evidence of clonal evolution with respect to *NRAS, KRAS, BRAF, PIK3A* or *EGFR* associated with this patient's disease progression on PCD with *KRAS* (p.A146V) AF in tumour tissue and cfDNA of 8.0% and 8.0% and *BRAF* (p.D594G) AF of 15% and 7.9%, respectively.

Patient MYL-018: A 67-year old female with multiply relapsed MM had a *KRAS* (p.A146T) mutation in tumour tissue and cfDNA with an AF of 22% and 2.0% at screening for the PHL-9460 clinical trial. This patient initially responded to trametinib treatment with a decrease in the M spike and kappa light chains from 25.1 to 18.1 g L^{-1} and 4311 to 2563 mg L^{-1}, respectively. After 6 months on study protocol the patient progressed. After discontinuation of trametinib, there was a rapid increase of the M spike, free kappa light chain and cfDNA AF to 23.7 g L^{-1}, 13106 mg L^{-1}, and 6.3% respectively. The patient declined to have a bone marrow done at this time point.

Patient MYL-022: A 70-year old female with IgG kappa myeloma initially treated with bortezomib-based induction and autologous stem cell transplantation, followed by lenalidomide maintenance, then escalated to lenalidomide-dexamethasone followed by carfilzomib-pomalidomide-dexamethasone upon progression. She was next enrolled onto the OSI-906 clinical trial of bortezomib-dexamethasone-linsitinib (IGF-1 receptor inhibitor) receiving 9 cycles before progressing on treatment. The first cfDNA and BM samples were drawn pre-cycle 2 of bortezomib-dexamethasone-linsitinib therapy while the second set of samples was drawn at the time of discontinuation from study. This second set of samples was also used as screening for study entry into the PHL-9460 clinical trial of trametinib. She was enrolled in PHL-9460 trial 4 years after initial diagnosis. She had stable disease after five cycles of trametinib at which time AKT inhibitor was added to trametinib, as per protocol. At the time of last follow-up, she was continuing on trial. Her *NRAS* p.Q61R allelic fraction in both cfDNA and BM increased from cycle 2 to cycle 9 of bortezomib-dexamethasone-linsitinib treatment coincident with an increase in her free kappa light chains and kappa/lambda ratio indicating clinical progression.

Patient MYL-027: A 54-year old female with multiply relapsed myeloma had *MLH1* (p.D394N), *KIT* (p.M552_Y553del), *TP53* (p.R213*) and *EGFR* (p.E551K) mutations in tumour tissue with AFs 19, 10, 12, and 14%, respectively. No mutations in *NRAS, KRAS, BRAF* or *PIK3CA* were detected in either tumour or cfDNA; however, *EGFR* p.E551K mutation was identified in the cfDNA with an AF of 44%. This patient had a very aggressive clinical course progressing within 3 months of high-dose chemotherapy and stem cell transplant with extramedullary disease as evidenced with marked splenomegaly. After failing treatment with pomalidomide, bortezomib, and dexamethasone, she was enrolled onto a clinical trial of selinexor (an inhibitor of XPO-1) and experienced a dramatic response with marked decrease in free light chains and reduction in spleen size. Unfortunately, the response was short lived and within 3 months she had progressed with increasing splenomegaly and free lambda light chains. At the time of progression, the tumour mutation profile demonstrated mutations in *MLH1, KIT, TP53,* and *EGFR* with AFs of 65, 89, 60 and 55%, respectively. Correspondingly, the AF of *EGFR* mutation detected in cfDNA was 47%.

Patient MYL-033: A 79-year old female with multiply relapsed MM had no evidence of mutations in *NRAS, KRAS, BRAF, PIK3CA* or *EGFR* in tumour tissue or cfDNA. She was stratified to the biomarker-negative arm of the PHL-9460. Her free lambda at the screening measured 891 mg L^{-1}. After 3 months on trametinib treatment the lambda light chains remained stable at 881 mg L^{-1} and no clonal evolution with respect to *NRAS, KRAS, BRAF, PIK3A*, or *EGFR* was observed in cfDNA analysis.

Patient MYL-043: A 61-year old male with multiply relapsed MM had a *KRAS* (p.G13D) mutation in tumour and cfDNA with an AF of 8.6% and 19%, respectively. This patient was stratified to the biomarker positive arm of the PHL-9460 clinical trial. Prior to starting treatment with trametinib, the serum free kappa level was 2610 mg L^{-1}. After one month on trametinib the patient was showing evidence of disease progression with an increase in the free kappa light chains to 5633 mg L^{-1} and evidence of bony and extramedullary progression that required radiotherapy treatment. Despite the clinical and biochemical progression, the AF of *KRAS* (p.G13D) in cfDNA decreased to 7.6% suggesting that trametinib was repressing the *KRAS* mutated clones but had little effect against the non-mutated tumour population. According to the protocol the AKT inhibitor GSK2141795 was added to trametinib at progression. Again, after one month of treatment with both trametinib and GSK2141795 the patient demonstrated discordant response with a decrease in the kappa light chains to 1935 g mL^{-1} and a new extramedullary lesion compressing the common bile duct. Again, we observed a decrease in the tumour and cfDNA AF of *KRAS* mutation to 1.2 and 1.5%, respectively suggesting that trametinib effectively controlled the *KRAS* mutated clone but had little effect against the bulk of the tumour cell population and evolution of extramedullary disease.

Patient MYL-049: A 53 year-old male with multiply relapsed MM had a *PIK3CA* (p.H59P) mutation detected in tumour and cfDNA with AFs of 41% and 40% respectively. In addition a *PIK3CA* (p.E545K) mutation was detected with an AF of 1.0% in the cfDNA only. This patient had evidence of extramedullary disease at the time of blood and BM sampling with lesions in liver, adrenal gland and pancreatic tail and we speculate that one or more of these lesions may have harboured the *PIK3CA* (p.E545K) that was not picked up in the BM tumour sample. No mutations were identified in *NRAS, KRAS*, or *BRAF* and thus the patient was stratified to the biomarker-negative arm of the PHL-9460 clinical trial. After one month of trametinib, the free kappa light chains had risen from 601.5 to 11947 mg mL^{-1} and the patient was taken off study. The AFs for *PIK3CA* p.E545K mutation detected in cfDNA and BM taken 6 weeks after initial sampling remained relatively stable, 38% and 36%, respectively, while the *PIK3CA* p.H59P mutation was again only detected in the blood with an AF of 2.1% (an increase from 1.0%).

Supplementary References

1. McKenna A, Hanna M, Banks E, et al. The Genome Analysis Toolkit: a MapReduce framework for analyzing next-generation DNA sequencing data. *Genome Res.* 2010;20(9):1297–1303.
2. The Broad Institute. Picard Tools, version 1.135. The Broad Institute; 2015.
3. Mulligan G, Mitsiades C, Bryant B, et al. Gene expression profiling and correlation with outcome in clinical trials of the proteasome inhibitor bortezomib. *Blood*. 2007;109(8):3177–3188.
4. Heitzer E, Ulz P, Geigl JB. Circulating tumor DNA as a liquid biopsy for cancer. *Clin. Chem.* 2015;61(1):112–123.
5. Heitzer E, Auer M, Hoffmann EM, et al. Establishment of tumor-specific copy number alterations from plasma DNA of patients with cancer. *Int. J. Cancer*. 2013;133(2):346–356.
6. Newman AM, Lovejoy AF, Klass DM, et al. Integrated digital error suppression for improved detection of circulating tumor DNA. *Nat. Biotechnol.* 2016;34(5):547–555.
7. Schmitt MW, Kennedy SR, Salk JJ, et al. Detection of ultra-rare mutations by next-generation sequencing. *Proc. Natl. Acad. Sci. U. S. A.* 2012;109(36):14508–14513.
8. Kennedy SR, Schmitt MW, Fox EJ, et al. Detecting ultralow-frequency mutations by Duplex Sequencing. *Nat. Protoc.* 2014;9(11):2586–2606.
9. Quail MA, Otto TD, Gu Y, et al. Optimal enzymes for amplifying sequencing libraries. *Nat. Methods*. 2011;9(1):10–11.
10. Minoche AE, Dohm JC, Himmelbauer H. Evaluation of genomic high-throughput sequencing data generated on Illumina HiSeq and genome analyzer systems. *Genome Biol.* 2011;12(11):R112.
11. Li H, Durbin R. Fast and accurate short read alignment with Burrows-Wheeler transform. *Bioinforma. Oxf. Engl.* 2009;25(14):1754–1760.
12. Cibulskis K, Lawrence MS, Carter SL, et al. Sensitive detection of somatic point mutations in impure and heterogeneous cancer samples. *Nat. Biotechnol.* 2013;31(3):213–219.
13. Ramos AH, Lichtenstein L, Gupta M, et al. Oncotator: cancer variant annotation tool. *Hum. Mutat.* 2015;36(4):E2423–2429.
14. Newman AM, Bratman SV, To J, et al. An ultrasensitive method for quantitating circulating tumor DNA with broad patient coverage. *Nat. Med.* 2014;20(5):548–554.
15. Iglewicz B, Hoaglin DC. How to Detect and Handle Outliers. ASQC Quality Press; 1993.
16. Chung N, Zhang XD, Kreamer A, et al. Median absolute deviation to improve hit selection for genome-scale RNAi screens. *J. Biomol. Screen.* 2008;13(2):149–158.
17. Hao C, Wang L, Peng S, et al. Gene mutations in primary tumors and corresponding patient-derived xenografts derived from non-small cell lung cancer. *Cancer Lett.* 2015;357(1):179–185.
